# Microbiome Composition in Both Wild-Type and Disease Model Mice Is Heavily Influenced by Mouse Facility

**DOI:** 10.3389/fmicb.2018.01598

**Published:** 2018-07-20

**Authors:** Kristopher D. Parker, Shannon E. Albeke, Jason P. Gigley, Allan M. Goldstein, Naomi L. Ward

**Affiliations:** ^1^Department of Molecular Biology, University of Wyoming, Laramie, WY, United States; ^2^Wyoming Geographic Information Science Center, University of Wyoming, Laramie, WY, United States; ^3^Department of Pediatric Surgery, Massachusetts General Hospital, Harvard Medical School, Boston, MA, United States; ^4^Department of Botany, University of Wyoming, Laramie, WY, United States

**Keywords:** reproducibility, replication, mouse microbiome, enterocolitis, C57BL/6J, Hirschsprung disease

## Abstract

Murine models have become essential tools for understanding the complex interactions between gut microbes, their hosts, and disease. While many intra-facility factors are known to influence the structure of mouse microbiomes, the contribution of inter-facility variation to mouse microbiome composition, especially in the context of disease, remains under-investigated. We replicated microbiome experiments using identical mouse lines housed in two separate animal facilities and report drastic differences in composition of microbiomes based upon animal facility of origin. We observed facility-specific microbiome signatures in the context of a disease model [the Ednrb (endothelin receptor type B) Hirschsprung disease mouse] and in normal C57BL/6J mice. Importantly, these facility differences were independent of cage, sex, or sequencing-related influence. In addition, we investigated the reproducibility of microbiome dysbiosis previously associated with Ednrb^-/-^ (knock-out; KO) mice. While we observed genotype-based differences in composition between wild-type (WT) and KO mice, these differences were inconsistent with the previously reported conclusions. Furthermore, the genotype-based differences were not identical across animal facilities. Despite this, through differential abundance testing, we identified several conserved candidate taxa and candidate operational taxonomic units that may play a role in disease promotion or protection. Overall, our findings raise the possibility that previously reported microbiome-disease associations from murine studies conducted in a single facility may be heavily influenced by facility-specific effects. More generally, these results provide a strong rationale for replication of mouse microbiome studies at multiple facilities, and for the meticulous collection of metadata that will allow the confounding effects of facility to be more specifically identified.

## Introduction

A growing body of evidence has revealed the substantial impact of gut microbes on maintaining health of the human host. Disruptions to healthy gut microbiomes have been associated with a wide variety of diseases, including metabolic (Ley et al., 2006), inflammatory (Swidsinski et al., 2002), and autoimmune diseases (Vaahtovuo et al., 2008), as well as cancer (Moore and Moore, 1995), mental illness (Cryan and Dinan, 2012), and developmental disorders (Ward et al., 2012; Kang et al., 2013).

Murine models dominate gut microbiome research due to their low cost, high reproductive rates, and ease of experimental manipulation. These manipulations are fundamental to investigating the potential causality in associations between dysbiosis and disease. Factors that influence murine gut microbiome variability and confound cross-study comparisons have been well documented within single animal facilities. Among these intra-facility factors, are mouse vendor and inter-individual variation (Deloris Alexander et al., 2006; Hildebrand et al., 2013; Ericsson et al., 2015), cage and mouse room effects (Deloris Alexander et al., 2006; Hildebrand et al., 2013), sex and genetic backgrounds (Deloris Alexander et al., 2006; Hufeldt et al., 2010; Kovacs et al., 2011), maternal effects and diet (Hildebrandt et al., 2009; Grönlund et al., 2011), and a wide range of stress responses and other environmental factors (Deloris Alexander et al., 2006; Bangsgaard Bendtsen et al., 2012). Infrastructure, technology, housing and husbandry practices, experimental protocols, diet, and other variables are likely to differ across animal facilities. The first systematic study to assess inter-facility effects documented a high-degree of facility-level individuality and variability in fecal microbiota of normal C57BL/6J mice across 21 facilities in Germany (Rausch et al., 2016). C57BL/6J mice provide the genetic background for many microbiome studies thus the findings of Rausch et al. (2016) are of great importance. To the best of our knowledge, there have been no reports describing the contribution of inter-facility effects to microbiome variability in the context of a diseased murine model.

Employing next-generation sequencing approaches, we compared gut microbiome structure and composition in a murine model of HSCR (Ednrb KO mouse) from two separate animal facilities. One of these facilities had been the location of a previous Hirschsprung study (Ward et al., 2012). HSCR is a congenital colorectal aganglionosis caused by failure of neural crest-derived cells to migrate into the distal portion of the colon. Up to 50% of HSCR patients develop a potentially fatal inflammatory colitis, HAEC (Burkardt et al., 2014). The etiology of HAEC remains unknown, hindering the ability to generate effective therapies for its prevention. Previous work using murine models of HSCR and HAEC revealed intestinal dysbiosis associated with aganglionosis, indicating a potential role for intestinal microbiomes in the promotion of HAEC (Ward et al., 2012; Pierre et al., 2014). We examined the reproducibility of these findings and also investigated the contribution of inter-facility effects to microbiome variability in C57BL/6J mice, reproducing the findings of Rausch et al. (2016) on a smaller scale.

## Results

### Facility-Specific Differences Are Major Contributors to Mouse Microbiome Composition in a Disease Model

**FIGURE 1:**
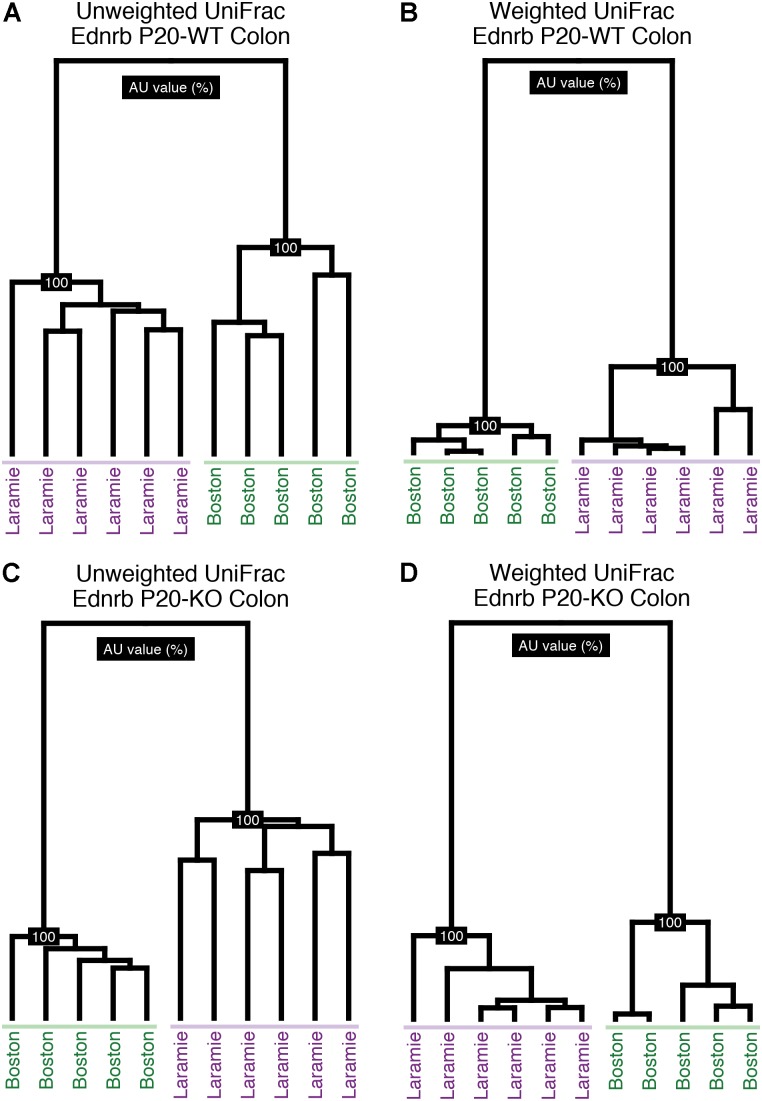
Colon samples from P20 Ednrb mice cluster by facility of origin. Hierarchical clustering of unweighted **(A,C)** and weighted **(B,D)** UniFrac distances for sequences obtained from P20-WT and P20-KO colon samples. Shading emphasizes each facility’s cluster.

We performed pairwise comparisons of PERMANOVA of UniFrac distances from Ednrb mice raised in Boston, MA, United States or Laramie, WY, United States. Statistical analysis revealed significant differences associated with animal facility for both colon and fecal sample microbiomes (Supplementary Table S1), with the exception of two weighted UniFrac groupings: P24-KO (24-day-old mice, KO; Ednrb^-/-^) colon and P24-WT (Ednrb^+/+^) fecal microbiomes. Hierarchical clustering visually demonstrated the facility bifurcation of P20 (20-day-old mice) Boston and P20 Laramie colon samples (**Figure 1**). The same topology was seen for P07 (7-day-old mice) and P24 mice, with some inter-digitation at both ages (Supplementary Figures S1, S2). These observations indicated a strong facility-specific effect on the composition of Ednrb mouse microbiomes at all ages.

To identify potential confounding factors underlying the compositional differences attributed to facility, we tested for cage effects in the Laramie Ednrb mice. Metadata necessary to examine cage effects were not available for Boston Ednrb mice. Given the variability in litter sizes, littermates at each age ranged from single pairs to multiple pairs of mice. Hierarchical clustering of UniFrac distances revealed some cage-related similarity for P07 microbiomes (Supplementary Figure S3), while cage-related clustering was not present in microbiomes from older P20 and P24 mice (Supplementary Figures S4, S5).

**FIGURE 2:**
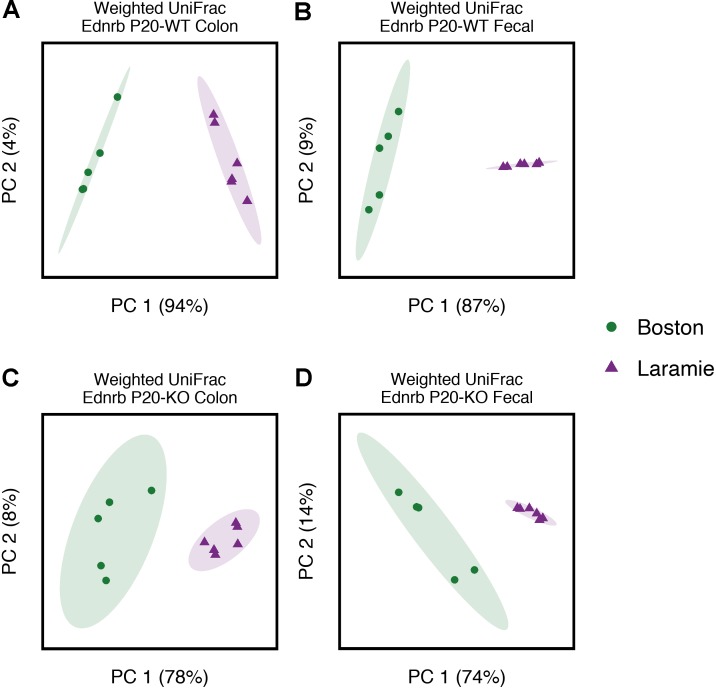
Core microbiomes of P20 Ednrb mice separate by facility of origin, with no overlap. Principal coordinates analysis of weighted UniFrac distances for core microbiome OTUs at the 50% threshold obtained from P20-WT colon **(A)** and fecal **(B)** samples, and P20-KO colon **(C)** and fecal **(D)** samples. Percentage values along each axis indicate the amount of variability in the data explained by each of the first two principal coordinates. Ellipses indicate 95% confidence intervals.

Given the facility differences reported above, we were interested in whether core microbiomes from each facility were also different. These analyses were performed for all groups on each facility individually and for both facilities together (“conserved core”). Conserved core OTUs represented less than half of the total number of OTUs for Ednrb mice at the 50% threshold (Supplementary Table S2). The number of conserved core OTUs dropped off considerably at the 75 and 100% thresholds, in many cases representing 0% of the total observed OTUs for a group. We calculated weighted UniFrac distances for the conserved core OTU tables and performed pairwise PERMANOVA to test for facility differences in the abundance of identical OTUs. Given minimal core conservation at the 75 and 100% thresholds, we utilized core OTUs at only the 50% threshold. No difference between facilities was observed for P24-KO colon or P24-WT fecal samples, while significant differences were observed for all other groups (Supplementary Table S1). Weighted UniFrac distances were ordinated using PCoA with 95% confidence ellipses around sample points for each facility (**Figure 2** and Supplementary Figures S6, S7). Ordination mostly corroborated the results of statistical analysis. P24-WT fecal and P24-KO colon samples exhibited no overlap of ellipses (Supplementary Figures S7B,C), despite statistical analysis revealing no support for facility-based differences within these groups (Supplementary Table S1). In addition, P24-WT colon samples showed some overlap (Supplementary Figure S7A), despite statistical support for facility-based differences between these groups (Supplementary Table S1). These observations highlight the general need for statistical analysis of group- or treatment-based differences in microbiome composition, rather than reliance on ordination or clustering approaches, even when 95% confidence intervals are used.

Ednrb mice were also assessed for facility-based microbiome differences using two taxonomy independent alpha diversity metrics: Chao1 and observed OTUs. For P07-WT colon samples, there was some statistical support for facility effects, although not consistently for both diversity metrics or both statistical tests (Supplementary Table S3). The same inconsistent trend was observed for P20-KO colon sample microbiomes. For the remaining groups, facility differences were not supported for either alpha diversity metric. These results suggest minimal contribution of facility-specific differences to the shaping of mouse microbiome diversity, as opposed to composition.

### Facility-Specific Differences Drive Microbiome Differences at All Taxonomic Levels

**FIGURE 3:**
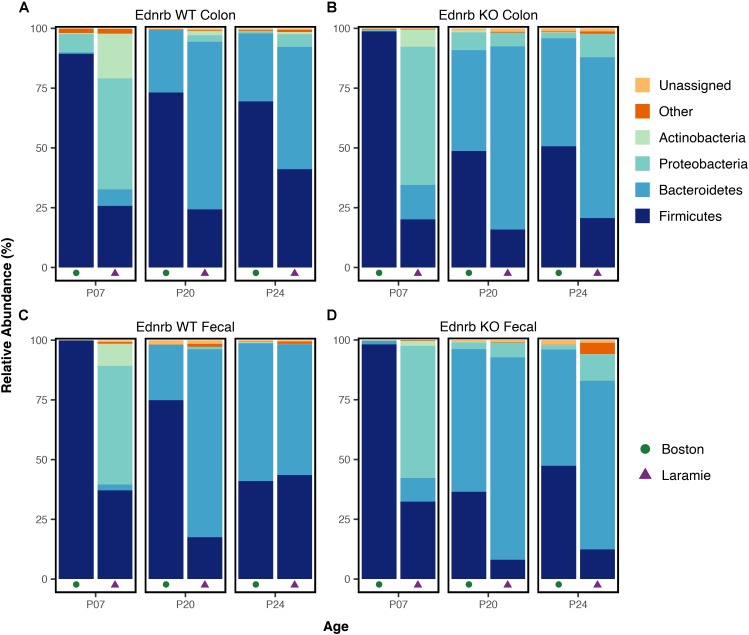
Phylum-level taxonomic composition of microbiomes from Ednrb mice exhibit marked differences in pattern between facilities. Mean relative abundances of four dominant phyla for samples grouped by facility, age, genotype, and sample type **(A–D)**. All taxonomic groups representing <6% of Bacterial sequences were grouped into “Other.” Any taxonomic group unable to be assigned to Kingdom Bacteria was grouped into “Unassigned.”

The compositional and abundance disparities between facilities in Ednrb mice were also evident when taxonomic information was considered. Mean relative abundances of dominant phyla showed marked and statistically supported differences in pattern when age- and genotype-matched mice were compared by facility (**Figure 3** and Supplementary Table S4). Firmicutes dominated P07-WT and P07-KO Boston mice, while P07-WT and P07-KO Laramie mice exhibited a more even composition of Firmicutes, Proteobacteria, and, as a smaller component, Actinobacteria. At P20, an enrichment of Bacteroidetes was observed in colon and fecal sample microbiomes in both facilities. In P24-WT fecal samples (**Figure 3C**), the phylum-level taxonomic composition from the two facilities appeared to converge. These mice showed similar patterns of abundance for Firmicutes and Bacteroidetes, while other mouse groups retained a P24 composition similar to their P20 composition (**Figures 3A,B,D**). At this age, there was no support for differences between facilities for any phyla (Supplementary Table S4).

We also tested for significant differences in relative abundance between sets of age- and genotype-matched mice from the two facilities, and report here only those taxa exhibiting a mean relative abundance of at least 6%. Taxonomy was assigned to the lowest rank possible. In both colon and fecal sample microbiomes, significant facility differences within a given mouse genotype were supported at all ages and every taxonomic level from order to genus (Supplementary Table S4). Taken together, the results of taxonomic assignment demonstrate facility-driven differences in taxon abundance at all classification levels, with mice from each facility demonstrating drastic differences in dominant taxa.

### Genotype-Based Differences in Diversity and Composition Are Not Consistent Across Facilities

We next asked whether previously reported genotype-based differences in beta diversity and in taxonomic composition between WT and KO Ednrb mouse microbiomes could be detected within each facility. We first re-assessed the Boston Ednrb mice, where the differences between WT and KO microbiomes (genotype-based differences) were originally reported (Ward et al., 2012). Analysis using pairwise Wilcoxon rank sum tests, but not pairwise Kruskal–Wallis, supported genotype-based differences in observed OTUs in P07 fecal samples and in Chao1 diversity in P20 colon samples (Supplementary Table S3). Comparisons of beta diversity between age-matched mice using pairwise PERMANOVA showed support for genotype-based differences for both sample types and both UniFrac metrics for P20 mice (Supplementary Table S5). A genotype difference in fecal samples from P07 mice was revealed for only the unweighted UniFrac metric. No differences were observed in P24 mice for either sample type or either beta diversity metric. Statistical analysis of mean relative abundances for taxa with greater than 6% abundance supported differences between genotypes in the abundance of phylum Bacteroidetes and family S24-7 in P20 fecal samples and family Enterobacteriaceae in P20 colon samples (Supplementary Table S6). These taxa were associated with P20-KO mice. Fecal samples from P07-WT mice possessed a higher abundance of Firmicutes. Taken together, these results indicate a strong genotype-based difference in microbiome composition between Boston P20 WT and KO mice, while a difference in P07 mice is suggested, but not as well supported.

We applied the same comparative approach to the Laramie Ednrb mice. Statistical analysis of alpha diversity supported no differences between genotypes at any age (Supplementary Table S3). Differences in microbiome composition based on unweighted (but not weighted) UniFrac distances were statistically supported for P24 mice in both sample types (Supplementary Table S5). Differences between genotypes at other ages were not supported. These results suggest that Laramie WT and KO P24 microbial communities differ in composition, but comparable patterns of OTU abundance drive these groups toward similarity. Comparisons of mean relative abundances for taxa above 6%, revealed genotype-based differences only in fecal sample microbiomes (Supplementary Table S6). Phylum Proteobacteria, family Enterobacteriaceae, and genera *Bacteroides* and *Parabacteroides* were associated with P24-KO mice. Enterobacteriaceae was also observed in higher abundance in P20-KO mice. Family S24-7 was associated with P20-WT and P24-WT mice, the inverse of what was observed in P20 Boston Ednrb mice.

### Boston and Laramie Facilities Share Patterns of Differential Abundance

We next attempted to identify organisms that may be responsible for contributing to or promoting colitis (associated with KO mice) or those that may serve a protective role (associated with WT mice). To assess this, we first determined which OTUs and taxa were differentially abundant in microbiomes of WT or KO mice for each facility separately. These results were combined and filtered to produce a list of conserved candidate OTUs or taxa associated with the same age and genotype across both facilities. A total of five candidate OTUs were discovered. One candidate was associated with P20- and P24-WT mice. Two candidates were associated with P24-WT mice, and two candidates were associated with P24-KO mice (**Table 1**). Candidate OTU-549756, representing *Lactobacillus* (**Table 1**), was also observed in P07 fecal samples; however, it did not exhibit a conserved genotype-association for both facilities at this age (Supplementary Table S7). We also identified three candidate taxa representing two taxonomic levels. Family S24-7 associated with P07-KO mice while family Enterobacteriaceae and genus *Coprobacillus* were both associated with P24-KO mice (**Table 1**). Family S24-7 was also observed in P20 fecal samples, although it shared an inverse facility relationship between genotypes (Supplementary Table S7).

**Table 1:** Differentially abundant candidate OTUs and taxa from Ednrb mice conserved between facilities.

OTU-ID	Taxonomic identity	Statistical test	Sample type	Association	Facility	Log_2_ FC
376516^∧^	O: Clostridiales	*g*-Test	Colon	P20-WT	Boston	−4.5
					Laramie	−10
376516^∧^	O: Clostridiales	*g*-Test	Colon	P24-WT	Boston	−10
					Laramie	−2.9
354957	O: Clostridiales	Kruskal–Wallis	Colon	P24-WT	Boston	−3.2
					Laramie	−10
549756	G: *Lactobacillus*	*g*-Test	Colon	P24-WT	Boston	−1.8
					Laramie	−10
351309	O: Clostridiales	*g*-Test	Colon	P24-KO	Boston	+3.8
					Laramie	+3.0
4407703	G: *Coprobacillus*	Kruskal–Wallis	Fecal	P24-KO	Boston	+5.5
					Laramie	+10
4407703	G: *Coprobacillus*	Kruskal–Wallis	Colon	P24-KO	Boston	+10
					Laramie	+3.8
n/a	F: S24-7	Non-parametric-T	Colon	P07-KO	Boston	+7.1
					Laramie	+1.8
n/a	F: Enterobacteriaceae	Kruskal–Wallis	Fecal	P24-KO	Boston	+3.2
					Laramie	+9.6
n/a	G: *Coprobacillus*	Kruskal–Wallis	Fecal	P24-KO	Boston	+5.6
					Laramie	+10
n/a	G: *Coprobacillus*	Non-parametric-T	Colon	P24-KO	Boston	+5.4
					Laramie	+2.2

*The symbol “^∧^” indicates OTUs or taxa observed at multiple ages. O, order; F, family; G, genus. Log_2_ fold change (FC) was calculated KO/WT, therefore “+” indicates association with KO mice and “-” indicates association with WT mice.*

### Facility-Specific Differences Are Major Contributors to Microbiome Composition in C57BL/6J Mice

Our observations in Ednrb mice together with a previous study in normal mice (Rausch et al., 2016), prompted us to determine whether facility effects also drive the structure of microbiomes in a normal in-bred mouse strain housed in our animal facilities.

**FIGURE 4:**
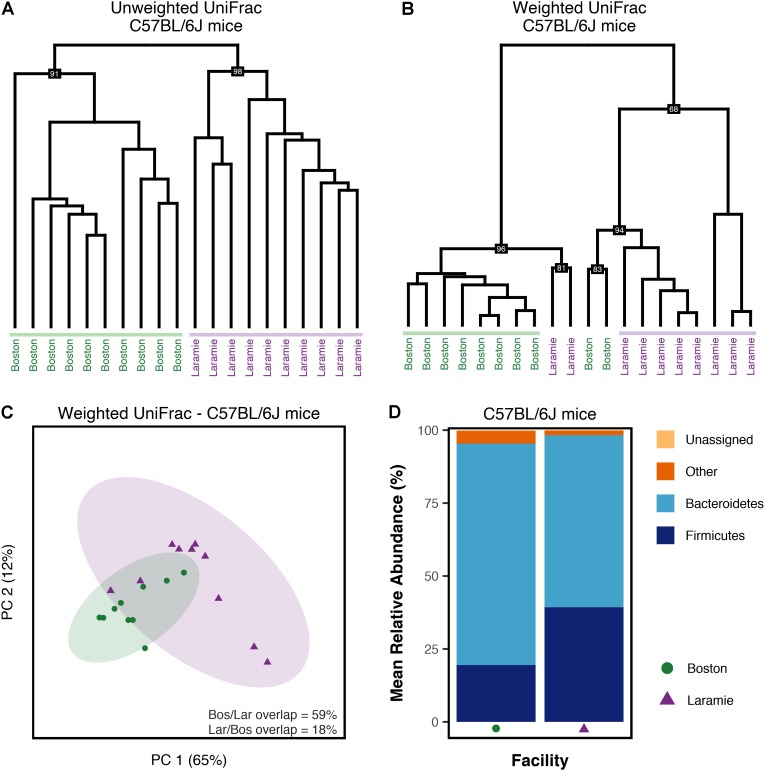
Microbiome composition of C57BL/6J mice differs by facility. **(A–D)** Analysis of sequence data from C57BL/6J mouse fecal samples from Boston and Laramie. **(A)** Hierarchical clustering of unweighted UniFrac distances. **(B)** Hierarchical clustering of weighted UniFrac distances. **(C)** Principal coordinates analysis of weighted UniFrac distances for core microbiome OTUs at the 50% threshold. Percentage values along each axis indicate the amount of variability in the data explained by each of the first two principal coordinates. Ellipses indicate 95% confidence intervals. Percentage of ellipse overlap is indicated. **(D)** Mean relative abundances of three dominant phyla for samples grouped by facility. All taxonomic groups representing <6% of Bacterial sequences were grouped into “Other.” Any taxonomic group unable to be assigned to Kingdom Bacteria was grouped into “Unassigned.”

PERMANOVA of UniFrac distances from C57BL/6J mice revealed significant differences in fecal microbiome composition associated with facility (Supplementary Table S1). Hierarchical clustering of UniFrac values provided a clear visualization of the facility effect (**Figures 4A,B**). Clusters derived from weighted UniFrac analysis (**Figure 4B**) displayed minor inter-digitation of each facility group; however, statistical testing supported a difference between these groups. Given reported sex and cage effects on microbiome composition (Deloris Alexander et al., 2006; Hildebrand et al., 2013), we looked at whether mice within a facility clustered according to these factors. There was some evidence for clustering by sex in both unweighted and weighted UniFrac analysis (Supplementary Figures S8A,B). In the cases where groups clustered by sex, they also clustered by cage. These cases were in the minority indicating a strong effect of facility on normal mouse microbiomes independent of sex or cage-related effects. Conserved core OTUs represented 54 and 22% of the total number of OTUs at the 50 and 75% thresholds, respectively (Supplementary Table S2). The percentage of conserved core OTUs was higher at all thresholds for C57BL/6J mice than for any group of Ednrb mice (Supplementary Table S2), although at the 100% threshold, the conserved core accounted for only 8% of the total observed OTUs. This suggests greater similarity in normal mouse microbiomes compared to microbiomes from diseased mice. The facility effect was statistically supported at the 50% core threshold (Supplementary Table S1). PCoA visually demonstrated this difference, while revealing overlap shared between Boston and Laramie ellipses (**Figure 4C**). No statistical support for differences in alpha diversity between facilities was found for C57BL/6J mice, using either Chao1 or observed OTUs metrics (Supplementary Table S3). Mean relative abundances of dominant phyla showed statistically supported differences between facilities (Supplementary Table S4). Boston mice were dominated by Bacteroidetes while a higher abundance of Firmicutes was observed in Laramie mice (**Figure 4D**).

## Discussion

In this study, we sampled colon and fecal microbiomes from two identical murine models housed in two separate animal facilities. These models included both an intestinal disease model (Ednrb), and one of the most widely used in-bred mouse strains (C57BL/6J). We identified a strong facility-specific effect on the composition of Ednrb mouse microbiomes in both genotypes and at all ages tested. The same facility-specific effect was also seen in fecal samples from C57BL/6J mice. These effects were independent of sex or cage influences. Our findings in C57BL/6J mice are consistent with a previous report (Rausch et al., 2016).

For both studies (HSCR and C57BL/6J), mouse facility practices and standards differed between Boston and Laramie in a number of ways, including diet, housing, and husbandry. In both humans and mice, the effects of diet on gut microbiomes have been well established (Brown et al., 2012). Little is known about the effects of different brands of standard mouse chow on the composition of gut microbiomes (Laukens et al., 2016); however, associations between mouse chow fat content and microbiome composition have been reported (Turnbaugh et al., 2008; Hildebrandt et al., 2009; Ma et al., 2012). Chow fed to Boston mice contained 12.3% fat, while Laramie chow contained 11.4% fat. In 11 out of 12 groups of age- and genotype-matched Ednrb mice, inter-facility comparisons showed a higher abundance of Firmicutes and a lower abundance of Bacteroidetes for Boston mice relative to Laramie mice. This relationship is a hallmark observation in mice fed high-fat diets (Hildebrandt et al., 2009; Brown et al., 2012). In contrast, the Boston C57BL/6J mice possessed lower Firmicutes and higher Bacteroidetes compared to Laramie mice. These observations suggest that the 0.9% difference in chow fat content may play a role separating microbial communities from the two facilities but is not the sole contributor to facility variation.

Treatment of mouse chow via autoclaving or irradiation is known to influence the physical properties and nutrient availability of chow (Caulfield et al., 2008). The use of irradiated chow has been associated with a decrease in microbiome diversity (Rausch et al., 2016); however, the factors underlying that association remain unclear. Our results showed minimal differences in alpha diversity between Boston mice, fed irradiated chow, and Laramie mice, fed autoclaved chow. Further studies that control for other confounding factors, including housing and husbandry discrepancies and mouse chow brand, could substantiate the possible link between irradiated chow and decreased alpha diversity. In addition, future experiments aimed at assessing the Boston and Laramie chow fed to mice in both facilities is warranted to determine whether diet alone is responsible for driving facility variation.

Intrinsic environmental factors, such as the interaction of mice with their cage mates, are known to influence the composition of gut microbiomes (Deloris Alexander et al., 2006; Kovacs et al., 2011; Hildebrand et al., 2013). Mice are known to ingest feces excreted by their co-housed mates either through direct consumption or through grooming (Soave and Brand, 1991). This practice, called coprophagy, is likely the primary driver of gut microbiota convergence or synchronization in co-housed mice (Deloris Alexander et al., 2006; Hildebrand et al., 2013; Nguyen et al., 2015). Transference of susceptibility to chemically induced colitis has been shown when WT mice are co-housed with mice deficient in immune system components, and is likely a result of transmission of colitogenic microbiota through coprophagy (Elinav et al., 2011; Zenewicz et al., 2013). For these reasons, our mice were housed as littermates where applicable. While metadata for Boston Ednrb mice were limited, precluding a cage effect analysis, the Ednrb Laramie mice showed minimal evidence for cage-related effects. Mice in our C57BL/6J study also showed minimal evidence for cage contribution to the shaping of microbiome composition. In both mouse lines analyzed in this study, cage effects are unlikely to be the primary driver of facility differences reported here, although in combination with other facility-specific factors may help drive microbial communities apart.

The previously reported (Ward et al., 2012) differences between WT and KO microbiomes were less clear after the Ednrb Boston mouse data were re-analyzed. This is likely attributable to the increased stringency of our re-analysis and the addition of rarefaction. Results from our replication study (Ednrb Laramie) showed partial reproducibility of genotype-based differences in colon and fecal microbiomes between WT and KO mice. Nonetheless, these differences and the overall microbiome composition and structure were not identical in both animal facilities. Phylum-level relative abundances in Boston Ednrb mice corroborated the original observation of increased Bacteroidetes in the feces of P20-KO mice, relative to WT mice of the same age. This relationship was not seen in Laramie Ednrb mice at this age. Instead, Laramie Ednrb mice showed an enrichment of family Enterobacteriaceae from phylum Proteobacteria. Observations for these taxa are consistent with previously published work in another HSCR/HAEC mouse model (Pierre et al., 2014) and shifts in Bacteroidetes (Frank et al., 2007; Walker et al., 2011) and Proteobacteria (Frank et al., 2007) have been reported in other IBD related to HAEC.

Members of the genus *Bacteroides* have been previously suggested to play a role in IBD (Swidsinski et al., 2005). In the context of HAEC, Frykman et al. (2015) observed higher abundance of *Bacteroides* in fecal samples from patients with a history of HAEC compared to patients with no history of enterocolitis, although this relationship was only qualitative (Frykman et al., 2015). In contrast, Li et al. (2016) observed statistically supported higher abundance of *Bacteroides* in the intestinal contents of patients with no history of enterocolitis compared to patients with active enterocolitis or patients in HAEC remission (Li et al., 2016). This genus was previously observed in higher abundance in the cecal contents of KO-mice in a similar HSCR/HAEC model (Pierre et al., 2014). In our original analysis (Ward et al., 2012), statistical support for higher abundance of *Bacteroides* was observed in the colons of Boston Ednrb P24-KO mice compared to WT mice of the same age; however, this association was not statistically supported after re-analysis. Interestingly, Laramie P24-KO mice displayed a higher abundance of this genus which was statistically supported in fecal samples. Further investigation of *Bacteroides* in the context of HAEC is warranted to determine whether this genus possesses proinflammatory ability or contributes to development of enterocolitis.

A powerful advantage of replicating a study in a different location was the ability to construct a conserved candidate list of differentially abundant organisms (OTUs) and taxa that share the same relationship across facilities. We identified five candidate OTUs and three candidate taxa; however, we want to emphasize caution when interpreting these results, as further empirical study of each candidate will be needed.

One candidate taxon corresponding to family S24-7 was over-represented in P07-KO mice compared to WT mice of the same age. This taxon did not maintain candidacy at the later ages as it was associated with Boston KO and Laramie WT mice at P20, and was not conserved for either genotype at P24. Members of family S24-7 appear to be abundant in laboratory mice, but have also been found in other mammals, including humans (Lagkouvardos et al., 2016; Ormerod et al., 2016). Increased abundance of family S24-7 was associated with treatment-induced remission in a mouse model of colitis (Rooks et al., 2014), suggesting a possible role for members of this family in protection from colitis. The inconsistent genotype associations for this candidate and the relative abundance of this family (see above) likely preclude this candidate from influencing the pathogenesis of HAEC, although repeat studies are needed.

Three candidate OTUs were associated with older Ednrb WT mice. Two represented order Clostridiales and one represented genus *Lactobacillus*. Our inability to classify Clostridiales candidates below the order level (for both these WT associations, and the KO associations described below) precludes even a tentative proposal of these candidates as colitogenic or colitis-protective. A significant depletion of *Lactobacillus* was observed in stool obtained from patients with HAEC (Shen et al., 2009). Lower abundance of *Lactobacillus* was also observed in diseased mice compared to healthy mice in another HSCR/HAEC murine model (Pierre et al., 2014). In humans (Mattar et al., 2003; Thiagarajah et al., 2014) and in Ednrb^-/-^ mice (Thiagarajah et al., 2014; Yildiz et al., 2015), a decrease in intestinal mucin production and alterations to the mucosal barrier have been associated with HAEC. A reduction in the availability of mucins may hinder the ability of lactobacilli to colonize the intestinal barrier and displace pathogens, a protective function normally performed by these organisms (Lee et al., 2003). Our discovery of a *Lactobacillus* candidate OTU in P24-WT colon samples further highlights the possible protective role of lactobacilli in prevention of HAEC.

We also identified two candidate OTUs and two candidate taxa associated with Ednrb P24-KO mice. The candidate OTUs corresponded to genus *Coprobacillus* and order Clostridiales, while the candidate taxa were identified as genus *Coprobacillus* and family Enterobacteriaceae. Enrichment of *Coprobacillus* has been observed in patients suffering PSC with concomitant IBD (Bajer et al., 2017). In contrast to these observations, *Coprobacillus* was found to be restricted to healthy patients rather than those with active CD (Rausch et al., 2011). This genus has also been associated with irritable bowel syndrome (Kassinen et al., 2007). The contradictory associations for *Coprobacillus* may be attributed to the observation of this genus as a member of the core microbiome of the gut in humans (Tap et al., 2009). Our observation of *Coprobacillus* as both a candidate OTU and a candidate taxon found in colon and fecal samples of P24-KO mice, a time point just prior to onset of HAEC, suggests a role for this organism in promoting inflammation. Increased abundance of Enterobacteriaceae is considered a marker of intestinal inflammation and oxidative stress in human IBD and in murine colitis (Jia et al., 2017). In the context of existing literature, our observation of this taxon in the feces of P24-KO mice from both facilities suggests a potentially prominent role for members of this family in promoting HAEC. Although short, our conserved candidate list provides targets for investigation of the contribution of specific organisms to protection from, or promotion of, HAEC.

## Conclusion

Our findings highlight the major effect that inter-facility variation has on microbiome composition both in a specific disease model and also in a mouse strain widely used in microbiome research. These results emphasize the need to exercise caution in the interpretation of microbiome-disease associations identified through single-facility murine studies. Our study was limited by inclusion of only two facilities, relatively low numbers of mice, and differences in available metadata from each facility. Identification of inter-facility differences through meticulous collection of metadata are essential to improve our understanding of the role that facility-specific factors play in shaping microbiomes and thereby diminish the variables that confound cross-study comparisons of murine microbiome work. Additionally, conducting microbiome studies in multiple facilities will strengthen the results obtained by permitting identification of shared taxa that can be further investigated as disease-associated candidates.

## Materials and Methods

### Ethics Statement

The Institutional Animal Care and Use Committees (IACUC) of the Massachusetts General Hospital for Children, and the University of Wyoming, approved all mouse experiments conducted in Boston, MA (C57BL/6J study and Hirschsprung Disease Boston study) and Laramie, WY (C57BL/6J study and Hirschsprung Disease Laramie study), respectively.

### Animal Facility Procedures

C57BL/6J or Ednrb^tm1Y wa^ mice on a hybrid C57BL/6J-129Sv background (JAX #003295) were housed under identical conditions within a given facility. A breeding pair from the Boston facility was used to establish the colony in Laramie. For both facilities, mice were maintained on a 12-h light–dark cycle at 25°C, supplied with autoclaved, hyper-acidified water (pH 3.0), and weaned at P21. Homozygous KO mice (Ednrb^-/-^) were phenotypically identified by their piebald coat, and genotype was confirmed by PCR as detailed in Section “Supplementary Methods” in Supplementary Data Sheet 1. Differences in mouse housing, diet, and husbandry between the Boston and Laramie facilities are listed below.

#### Boston Housing, Diet, and Husbandry

Mice were housed in Allentown rectangular cages (Allentown Inc., Allentown, NJ, United States) and cage/bedding changes were performed weekly or as needed. Mice were fed a standard non-autoclaved rodent chow, Prolab Isopro RMH 3000 Irradiated (LabDiet, St. Louis, MO, United States). Mice were harem bred (one male and two females per cage).

#### Laramie Housing, Diet, and Husbandry

Mice were housed in Optimice polysulfone triangular cages (Animal Care Systems, Centennial, CO, United States) and cage/bedding changes were performed every 2 weeks or as needed. Mice were fed a standard, autoclaved rodent chow, Laboratory Rodent Diet 5001 (LabDiet, St. Louis, MO, United States). Mice were monogamously bred (one male and one female per cage), as this was the only breeding protocol approved by the University of Wyoming IACUC.

### Sample Collection and DNA Extraction

#### Hirschsprung Disease Studies (Boston Ednrb and Laramie Ednrb)

Knockout (Ednrb^−/−^) and WT (Ednrb^+/+^) mice were euthanized by CO_2_ asphyxiation on P07, P20, and P24. The sampling time points chosen encompassed both weaning and disease progression to capture the environment prior to disease. At P07 and P20, mice are suckling, with P20 mice having additional access to solid food. P24 mice are completely weaned and near the usual onset time of HAEC (P28-P30). The distal two-thirds of each mouse colon was removed and rinsed with sterile phosphate-buffered saline (PBS) (pH 7.4). Colons were collected for analysis of surface-associated microbial communities. Fecal material was collected from the colon washout. Colons and fecal material were immediately frozen at -80°C.

#### C57BL/6J Study

Fecal samples were collected when mice were 6 weeks of age. Pellets were collected during defecation directly into sterile cryotubes pre-filled with 0.5 mL 1× PBS (pH 7.4) supplemented with 10% glycerol. Material was collected from 10 mice at each facility, three females and seven males. Mice were housed by both litter and sex. DNA was extracted using the QIAmp DNA stool MiniKit (QIAGEN, Valencia, CA, United States) with the addition of a bead beating step at the beginning. Full details are available in the Section “Supplementary Methods” in Supplementary Data Sheet 1.

#### Sample Numbers

In the originally published Boston Ednrb study (Ward et al., 2012), statistical support for a significant differences in microbiome composition and structure between Ednrb^-/-^ (KO) and Ednrb^+/+^ (WT) mice were observed using *n* = 5/genotype at P07 and P20, and *n* = 3/genotype at P24. Our replication study (Ednrb Laramie) used similar numbers: *n* = 5/genotype at P07 and P24, and *n* = 6/genotype at P20. Two mice per genotype from the original Laramie P24 cohort sequenced poorly, and an additional two mice per genotype were sampled to maintain *n* = 5/genotype. The C57BL/6J dataset used *n* = 10 mice/facility. In a methodologically similar study comparing fecal microbiomes of C57BL/6J mice from several animal facilities, Rausch et al. (2016) were able to make statistically meaningful and biologically relevant conclusions based upon *n* = 5 mice/facility. Other studies using smaller numbers of animals (*n* = 4/group) (Kovacs et al., 2011) or similar numbers (*n* = 7–10/group) were able to demonstrate statistical support for group-based differences (Shogan et al., 2014; Allen et al., 2015; Ryan et al., 2017). Based upon these published studies, it was reasonable to expect that the work performed here was adequately powered.

### 16S rRNA Gene Sequencing – Roche 454 and Illumina MiSeq

Roche 454 pyrosequencing utilized the 28F-588R primer pair targeting the V1–V3 hypervariable 16S rRNA regions and Illumina MiSeq sequencing utilized the primer pair 28F-388R targeting the V1–V2 hypervariable regions. Samples from the HSCR studies were sequenced at different times: 2012 for Boston Ednrb and 2015 for Laramie Ednrb. Samples from the C57BL/6J study were sequenced at the same time, in the same lane. Resamples from P24 Laramie Ednrb mice were sequenced at the same time, in the same lane. Detailed sequencing methods are available in the Section “Supplementary Methods” in Supplementary Data Sheet 1.

### Sequence Processing

Quality trimming, chimera checking, and denoising of raw datasets was performed by RTL (see section “Supplementary Methods” in Supplementary Data Sheet 1 for full parameters and details). Pre-processed sequencing files obtained from RTL were further processed and analyzed using the QIIME 1 pipeline (Caporaso et al., 2010b). We analyzed our replication study (Ednrb Laramie) alongside the original study (Ednrb Boston) to ensure consistency across all parameters used for data processing and analysis. For all datasets, sequence reads were first demultiplexed and then quality filtered. Quality filtering removed any reads not matching the sample-specific barcode, reads shorter than 200 bp or longer than 1000 bp, reads with greater than six ambiguous bases, reads below the minimum quality score of 25, and reads with homopolymers in excess of 6 bp. Individual sequence files for HSCR-Boston and HSCR-Laramie were merged following demultiplexing and quality filtering. Sequences were classified into OTUs, defined at 97% 16S rRNA gene sequence similarity, and selected using UCLUST open-reference clustering against Greengenes reference collection 13.8 (DeSantis et al., 2006; Edgar, 2010). UCLUST was chosen over USEARCH as chimeric sequences were removed by RTL during the pre-processing steps. Reads that failed to cluster to a reference were subsequently clustered *de novo*. Representative sequences from each OTU were aligned to a core set of Greengenes 16S rRNA sequences using PyNAST and subsequently filtered (DeSantis et al., 2006; Caporaso et al., 2010a). Phylogenetic trees relating OTUs were constructed using FastTree (Price et al., 2010). Taxonomy for each representative OTU was assigned against Greengenes 13.8 using UCLUST consensus taxonomy (Edgar, 2010; McDonald et al., 2012). Finally, chloroplast sequences were removed from each OTU table using QIIME 1. The above workflow produced an unrarefied master OTU table with taxonomic assignments for each OTU and containing all samples within a given study. Samples from each unrarefied master OTU table were filtered out as needed for subsequent analysis, yielding separate OTU tables. The fates of each of these tables are and their rarefaction depths are detailed in Section “Supplementary Methods” in Supplementary Data Sheet 1 and “Supplementary Table S8,” respectively.

### OTU Table Analyses

Beta diversity was quantified using unweighted and weighted UniFrac distance metrics (Lozupone and Knight, 2005). Unweighted UniFrac considers only presence/absence of an OTU, whereas weighted UniFrac takes into account the relative abundance of an OTU in addition to presence/absence. UniFrac distances were visualized with either hierarchical clustering using UPGMA (Sokal and Michener, 1958) or with ordination utilizing PCoA (Borg and Groenen, 2005). UPGMA clustering was multiscale bootstrap resampled in R using package pvclust (Suzuki and Shimodaira, 2006). Bootstrapping was performed *n* = 10,000 replications to calculate approximately unbiased (AU) percentage values for each cluster and build a statistically supported consensus dendrogram. AU-values 95% and above indicate strong support for branches. Dendrograms were visualized in R using the base packages in combination with dendextend, dplyr, ggplot2, and gridExtra (Wickham, 2009; Galili, 2015; Auguie, 2016; R Core Team, 2017; Wickham et al., 2017). Principal coordinates were calculated in R using package ape (Paradis et al., 2004), and illustrated using R packages rKIN and ggplot2 (Wickham, 2009; Albeke, 2017). Package rKIN was also used to compute the percentage of overlap between 95% confidence ellipses in PCoA ordination. The species richness (alpha diversity) of samples was measured in QIIME 1 using both the Chao1 and observed OTUs metrics. The Chao1 index estimates the total number of distinct OTUs in a sample, while the observed OTUs metric measures the actual observed number of distinct OTUs per sample. Core microbiomes were computed using QIIME 1, and were defined as OTUs present in 50, 75, or 100% of samples within a grouping, allowing for comparisons at multiple percentage thresholds. Phylum- and genus-level relative abundances were extracted from OTU tables using QIIME 1. Phylum-level relative abundance plots were generated in R using a combination of dplyr, ggplot2, and gridExtra (Wickham, 2009; Auguie, 2016; Wickham et al., 2017).

### Statistical Analyses

Differences in UniFrac distances between sample groups were assessed using the PERMANOVA method, adonis, from R package vegan (Oksanen et al., 2017). Pairwise multiple comparisons were made for all possible combinations within a given distance matrix, and the resulting *P*-values were adjusted for FDR (Benjamini and Hochberg, 1995). Pairwise comparisons were calculated using an in-house R function (pwise.adon), which can be found in Supplementary Data Sheet 2. Differential abundance testing of OTUs and taxa on age-matched WT and KO mice from each facility was carried out in QIIME 1 using the Kruskal–Wallis, non-parametric *T*, and *g*-tests on rarefied OTU tables. The outcome from each test was variable, therefore all results were reported for transparency. To assess differences in alpha diversity or relative abundance of taxa between sample groups, two non-parametric rank sums tests were employed, Kruskal–Wallis and Wilcoxon (Toutenburg, 1975). Where applicable, Kruskal–Wallis was followed by Dunn’s *post hoc* analysis for pairwise multiple comparisons using R packages stats and PMCMR, respectively (Pohlert, 2014; R Core Team, 2017). R packages dplyr and reshape2 were used to construct Supplementary Tables S3, S4, S6 (Wickham, 2007; Wickham et al., 2017). Where applicable, the Wilcoxon test, also referred to as Mann–Whitney *U*, was followed by pairwise Wilcoxon comparisons between groups using R stats (R Core Team, 2017). Both multiple comparisons tests used the FDR method to adjust *P*-values (Benjamini and Hochberg, 1995). Pairwise comparisons were conducted for all possible pair combinations of all ages and all genotypes. These approaches yielded slightly different results. For transparency, we chose to report the results from both statistical methods to ensure that conclusions were not made based upon a single statistical test.

### Computational Details

Computational analyses were performed using QIIME 1 version 1.9.1, R Version 3.5.0 “Joy in Playing,” R Studio version 1.1.447, conda version 4.5.2, Python version 3.5.5, running on macOS High Sierra version 10.13.4.

## Availability of Data and Materials

The amplicon sequence datasets supporting the conclusions of this manuscript are available under the NCBI BioProject ID PRJNA418574. The QIIME 1 commands, OTU tables, accompanying metadata, R scripts and workspaces used for data processing, analysis, and visualization are available in Supplementary Data Sheet 2. File names, types, and descriptions for everything found in Supplementary Data Sheet 2 are located on the last page of Supplementary Data Sheet 1. All raw, filtered, and rarefied OTU tables, in addition to all commands, shell scripts, Jupyter notebooks, R scripts, and all other items generated and used during analysis are available in this project’s GitHub repository located here: https://github.com/kdprkr/WardsWizard. Please contact the corresponding author if any item needed for analysis or generation of figures and tables cannot be found, or if any links are broken.

## Author Contributions

JG, AG, and NW acquired funding and designed the approaches used for the HSCR studies. KP designed the approaches for the C57BL/6J study, performed all Laramie-based experiments, coded all bioinformatic and statistical analyses, visualized and interpreted the data, and wrote the manuscript. SA was sensei for initial coding work and provided direction for statistical analysis. JG provided guidance for initial mouse husbandry in Laramie. All authors read, reviewed, and approved the manuscript prior to submission.

## Conflict of Interest Statement

The authors declare that the research was conducted in the absence of any commercial or financial relationships that could be construed as a potential conflict of interest.

## Abbreviations

CD: Crohn’s disease
Ednrb: endothelin receptor type B
FDR: false discovery rate
HAEC: Hirschsprung-associated enterocolitis
HSCR: Hirschsprung disease
IBD: inflammatory bowel disease
KO: knock-out
OTUs: operational taxonomic units
P07: postnatal day 7
P20: postnatal day 20
P24: postnatal day 24
PCoA: principal coordinates analysis
PCR: polymerase chain reaction
PERMANOVA: permutational multivariate analysis of variance
PSC: primary sclerosing cholangitis
QIIME 1: Quantitative Insights Into Microbial Ecology 1
UPGMA: unweighted pair group method with arithmetic mean
WT: wild-type
