# Supplementary material for: Microbiome Composition in Both Wild-Type and Disease Model Mice Is Heavily Influenced by Mouse Facility
Source: Front Microbiol. 2018 Jul 20;9:1598. doi: 10.3389/fmicb.2018.01598 (PMC6062620; doi:10.3389/fmicb.2018.01598)
Supplement: Supplementary file 1 [file Data_Sheet_1.pdf]

Supplementary Materials: Microbiome composition in both wild-type and disease model mice is heavily influenced by mouse facility

Kristopher D. Parker[1], Shannon E. Albeke[2], Jason P. Gigley[1], Allan M. Goldstein[3], Naomi L. Ward[4*]

***Correspondence:**
Dr. Naomi L. Ward
nlward@uwyo.edu

SUPPLEMENTARY METHODS

Mouse genotyping

Mouse genomic DNA was extracted from ear tissue with either Red Extract-N-Amp Tissue PCR kit (Sigma-Aldrich, St. Louis, MO, USA) or Viagen DirectPCR Ear Lysis Reagent (Viagen Biotech, Los Angeles, CA, USA), using manufacturer protocols. For PCR, the following primer set was used: KO-forward: oIMR7770F ATAGATTCGCCCTTGTGTCC, WT-forward oIMR6449F GATGAACCTGCTCAGTGCAA, common reverse: oIMR6448R CATGGTCTTGTTCCTGATGC. Amplifications were performed in 25μL reactions with 1μL of each 10μM primer, 0.5μL 10mM dNTPs, 2μL 25mM $MgCl_2$, 0.125μL DNA polymerase, and 4μL template. Reactions were performed under the following thermal profile: 94°C for 3 min, then 35 cycles of 94°C for 30 sec, 60°C for 1 min, 72°C for 1 min, followed by one cycle of 72°C for 2 min and a 4°C hold. Amplification products were visualized using standard agarose gel electrophoresis. $Ednrb^{-/-}$ (KO) mice exhibit a fragment of 244bp. $Ednrb^{+/+}$ (WT) mice exhibit a fragment of 444bp. $Ednrb^{+/-}$ (heterozygous) mice exhibit fragments of 444bp and 244bp.

DNA extraction

Colon and fecal samples were thawed and DNA extraction was performed using the QIAmp DNA stool MiniKit according to manufacturer's instructions with the following modifications: fecal samples were vortexed at max speed for approximately one minute or until thoroughly homogenized in existing buffer. To colon samples, 600μL of sterile PBS (pH 7.4) was added, followed by vortexing at max speed for one minute. For both sample types, 600μL of supernatant was added to a 2mL tube prefilled with sterile 0.1mm diameter zirconium beads (Spectrum Scientifics, Philadelphia, PA). To each tube, 1mL buffer ASL was added. Samples were processed on a TissueLyzer II (QIAGEN, Valencia, CA, USA) for 5 minutes at 30Hz. Supernatants were removed following centrifugation at 13,000 x g for 1 minute. One tablet of InhibitEX (provided in the kit) was added to each to tube to absorb PCR inhibitors, followed by 5 minutes at 15Hz on the TissueLyzer II. Hereafter, samples were processed according to manufacturer instructions. Samples were eluted in 50μL buffer AE (pre-warmed to 50°C) provided in the kit.

16S rRNA gene sequencing – Roche 454

Samples from the HSCR studies were sequenced at different times: 2012 for Boston Ednrb and 2015 for Laramie Ednrb. Amplification and sequencing was performed by RTL Genomics, Lubbock, TX, USA. Samples were amplified for pyrosequencing using a forward and reverse

fusion primer. The forward primer was constructed with the Roche A linker: CCATCTCATCCCTGCGTGTCTCCGACTCAG, an 8bp barcode, and the 28F primer: GAGTTTGATCNTGGCTCAG. The reverse fusion primer was constructed with a biotin molecule, the Roche B linker: CCTATCCCCTGTGTGCCTTGGCAGTCTCAG, and the 519R primer: GTNTTACNGCGGCKGCTG. This primer pair corresponds to the V1-V3 hypervariable region of the 16S rRNA gene. Amplifications were performed in 25μL reactions with Qiagen HotStarTaq master mix (QIAGEN, Valencia, CA, USA), 1μL of each 5μM primer, and 1μL of template. Reactions were performed on ABI Veriti thermocyclers (Applied Biosytems, Carlsbad, CA, USA) under the following thermal profile: 95°C for 5 min, then 35 cycles of 94°C for 30 sec, 54°C for 40 sec, 72°C for 1 min, followed by one cycle of 72°C for 10 min and 4°C hold. Amplification products were visualized with eGels (Life Technologies, Grand Island, NY, USA). Products were then pooled equimolar and each pool was cleaned and size selected using Agencourt AMPure XP (BeckmanCoulter, Indianapolis, IN, USA) following Roche 454 protocols (454 Life Sciences, Branford, CT, USA). Size-selected pools were then quantified, diluted, and used in emPCR reactions, which were performed and subsequently enriched. Sequencing followed established manufacturer protocols (454 Life Sciences).

16S rRNA gene sequencing – Illumina MiSeq

Samples from the C57BL/6J study were sequenced at the same time, in the same lane. Resamples from P24 Laramie Ednrb mice were sequenced at the same time, in the same lane. Amplification and sequencing was performed by RTL Genomics, Lubbock, TX, USA. Samples were amplified for sequencing in a two-step process. The forward primer was constructed with the Illumina i5 sequencing primer: TCGTCGGCAGCGTCAGATGTGTATAAGAGACAG, and the 28F primer: GAGTTTGATCNTGGCTCAG. The reverse primer was constructed with the Illumina i7 sequencing primer: GTCTCGTGGGCTCGGAGATGTGTATAAGAGACAG, and the 388R primer: TGCTGCCTCCCGTAGGAGT. This primer pair corresponds to the V1-V2 hypervariable region of 16S rRNA. Amplifications were performed in 25μL reactions with Qiagen HotStarTaq master mix (QIAGEN, Valencia, CA, USA), 1μL of each 5μM primer, and 1μL of template. Reactions were performed on ABI Veriti thermocyclers (Applied Biosytems, Carlsbad, CA, USA) under the following thermal profile: 95°C for 5 min, then 25 cycles of 94°C for 30 sec, 54°C for 40 sec, 72°C for 1 min, followed by one cycle of 72°C for 10 min and 4°C hold. Products from the first-stage amplification were added to a second PCR based upon qualitatively determined concentrations. Primers for the second PCR were designed based on the Illumina Nextera PCR primers as follows: forward: AATGATACGGCGACCACCGAGATCTACAC-i5index-TCGTCGGCAGCGTC and reverse: CAAGCAGAAGACGGCATACGAGAT-i7index-GTCTCGTGGGCTCGG. The second-stage amplification was run under the same conditions as the first except for only 10 cycles. Amplification products were visualized with eGels (Life Technologies, Grand Island, NY, USA). Products were then pooled equimolar and each pool was size selected in two rounds using Agencourt AMPure XP (BeckmanCoulter, Indianapolis, IN, USA) in a 0.7 ratio for both rounds. Size-selected pools were then quantified using the Quibit 2.0 fluorometer (Life Technologies, Grand Island, NY, USA) and loaded on an Illumina MiSeq (Illumina, Inc. San Diego, CA, USA) 2x300 flow cell at 10pM.

Quality trimming, chimera checking, and denoising of raw datasets

These pre-processing steps improve the overall accuracy of sequences thus reducing the chance of falsely classified OTUs in downstream analyses. RTL performed pre-processing as follows: for

Illumina only, the forward and reverse reads were taken in FASTQ format and merged using the PEAR Illumina paired-end read merger (Zhang et al. 2014). For both Illumina and 454, the FASTQ-formatted files were converted into FASTA-formatted sequence and quality files. Reads were run through an internally developed quality-trimming algorithm. During this stage, a running average for each read was taken across the sequence and was trimmed back at the last base where the total average was greater than 25. Sequence reads were sorted by length from longest to shortest. Prefix dereplication was performed using the USEARCH algorithm (Edgar 2010). Briefly, prefix dereplication grouped reads into clusters such that each sequence of equal or shorter length to the centroid sequence must be a 100% match to the centroid sequence for the length of the sequence. Each cluster was marked with the total number of member sequences. Sequences less than 100bp in length were not written to the output file; however, no minimum cluster size restriction was applied, allowing singleton clusters to exist in the output. Following dereplication, clustering at 4% divergence was performed using the USEARCH clustering algorithm. The result of this stage was the consensus sequence from each new cluster, tagged to show its total number of member sequences (dereplicated + clustered). Clusters containing fewer than two members were not added to the output file, thus removing singletons from the dataset. Selection of Operational Taxonomic Units was performed using the UPARSE OTU selection algorithm to classify the large number of clusters into OTUs (Edgar 2013). Following OTU selection, chimera checking was performed using the UCHIME chimera detection software executed in *de novo* mode (Edgar et al. 2011). Each clustered centroid from USEARCH clustering was then mapped to its corresponding OTUs and marked as either chimeric or non-chimeric. All chimeric sequences were then removed. Each quality-trimmed read was then mapped to its corresponding non-chimeric cluster using the USEARCH global alignment algorithm. Using the consensus sequence for each centroid as a guide, each sequence in a cluster was then aligned to the consensus sequence and each base was corrected using the following rules where C was the consensus sequence and S was the aligned sequence: (1) if the current base pair in S was marked to be deleted, then the base was removed from the sequence if the quality score for that base was less than 30, (2) if the current position in S was marked to have a base from C inserted, then the base was inserted into the sequence if the mean quality score from all sequences that mark the base as existing was greater than 30, (3) if the current position in S was marked as a match to C, but the bases were different, then the base in S was changed if the quality score for that base was less than 30, (4) if a base was inserted or changed, the quality score for that position was updated, (5) if the base was deleted, the quality score for that position was removed, (6) otherwise, the base in S was left alone and correction moved to the next position. Finally, all of the corrected sequences were written to the output file in FASTA format.

OTU table fates and rarefaction depths

The unrarefied master OTU table (HSCR-OTU-table) was used for facility- or genotype-based comparisons of relative abundance of taxa. Prior to rarefaction, HSCR-OTU-table was split into separate OTU tables as follows: colon samples from both facilities (cBL-HSCR), fecal samples from both facilities (fBL-HSCR), all Boston colon samples (cBos-HSCR), all Boston fecal samples (fBos-HSCR), all Laramie colon samples (cLar-HSCR), all Laramie fecal samples (fLar-HSCR). Filtered OTU tables were individually rarefied to the depths listed in Table S8. Rarefaction creates a subsampled OTU table by randomly selecting equal numbers of sequences from each sample. This subsampling reduces the variability in sequencing depth between sequencing runs, while also reducing the disparity of depth between sequencing platforms. When

rarefying OTU tables, we recognize that valuable data may be lost from samples with higher coverage, especially when merging sequences from 454 and Illumina platforms. However, to apply statistical approaches and thus make meaningful conclusions, we needed the added samples from the P24 Laramie Ednrb Illumina resample. For these reasons, the 454 and Illumina sequence files from this resample were merged prior to OTU picking. Colon or fecal specific rarefied OTU tables (cBL-HSCR-rare and fBL-HSCR-rare) were used for inter-facility comparisons of alpha and beta, and core microbiome computation. We did not make inter-facility comparisons using OTU tables of different rarefaction depths. For intra-facility comparisons, individually rarefied OTU tables (cBos-HSCR-rare, fBos-HSCR-rare, cLar-HSCR-rare, and fLar-HSCR-rare) were used to assess genotype-based differences in alpha and beta diversity. For the C57BL/6J dataset, the unrarefied master OTU table (C57-OTU-table) was used for facility-based comparisons of relative abundance of taxa. The C57-OTU-table was rarefied to a depth of 12,790 and used for all other inter-facility comparisons.

REFERENCES FOR SUPPLEMENTARY METHODS

Edgar, Robert C. 2010. "Search and Clustering Orders of Magnitude Faster than BLAST." *Bioinformatics (Oxford, England)* 26 (19): 2460–61. https://doi.org/10.1093/bioinformatics/btq461.

———. 2013. "UPARSE: Highly Accurate OTU Sequences from Microbial Amplicon Reads." *Nature Methods* 10 (10): 996–98. https://doi.org/10.1038/nmeth.2604.

Edgar, Robert C., Brian J. Haas, Jose C. Clemente, Christopher Quince, and Rob Knight. 2011. "UCHIME Improves Sensitivity and Speed of Chimera Detection." *Bioinformatics* 27 (16): 2194–2200. https://doi.org/10.1093/bioinformatics/btr381.

Zhang, Jiajie, Kassian Kobert, Tomáš Flouri, and Alexandros Stamatakis. 2014. "PEAR: A Fast and Accurate Illumina Paired-End ReAd MergeR." *Bioinformatics (Oxford, England)* 30 (5): 614–20. https://doi.org/10.1093/bioinformatics/btt593.

SUPPLEMENTARY FIGURES

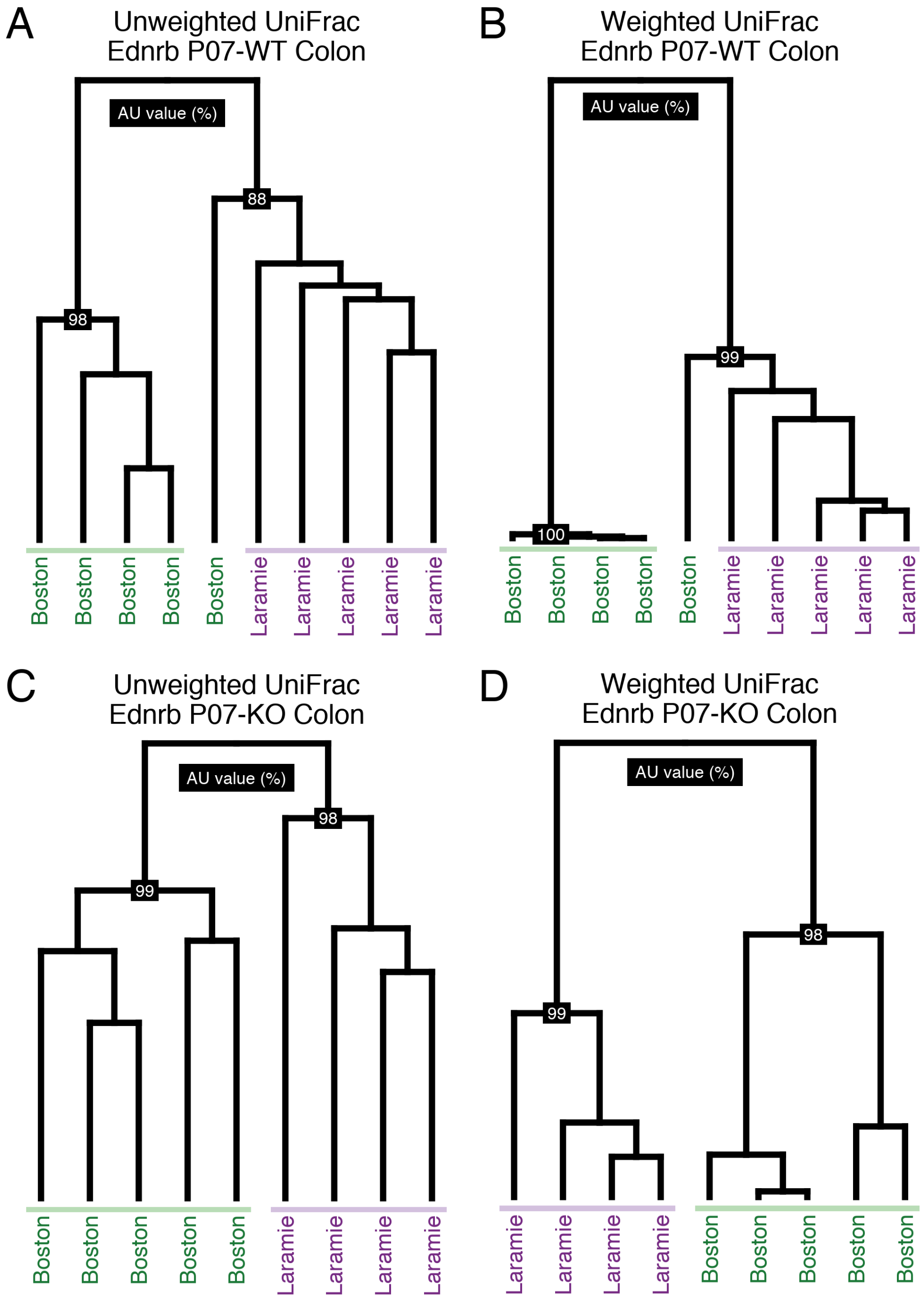

Figure S1 | Colon samples from P07 Ednrb mice cluster by facility of origin. Hierarchical clustering of unweighted (A, C) and weighted (B, D) UniFrac distances for sequences obtained from P07-WT and P07-KO colon samples. Shading emphasizes each facility's cluster.

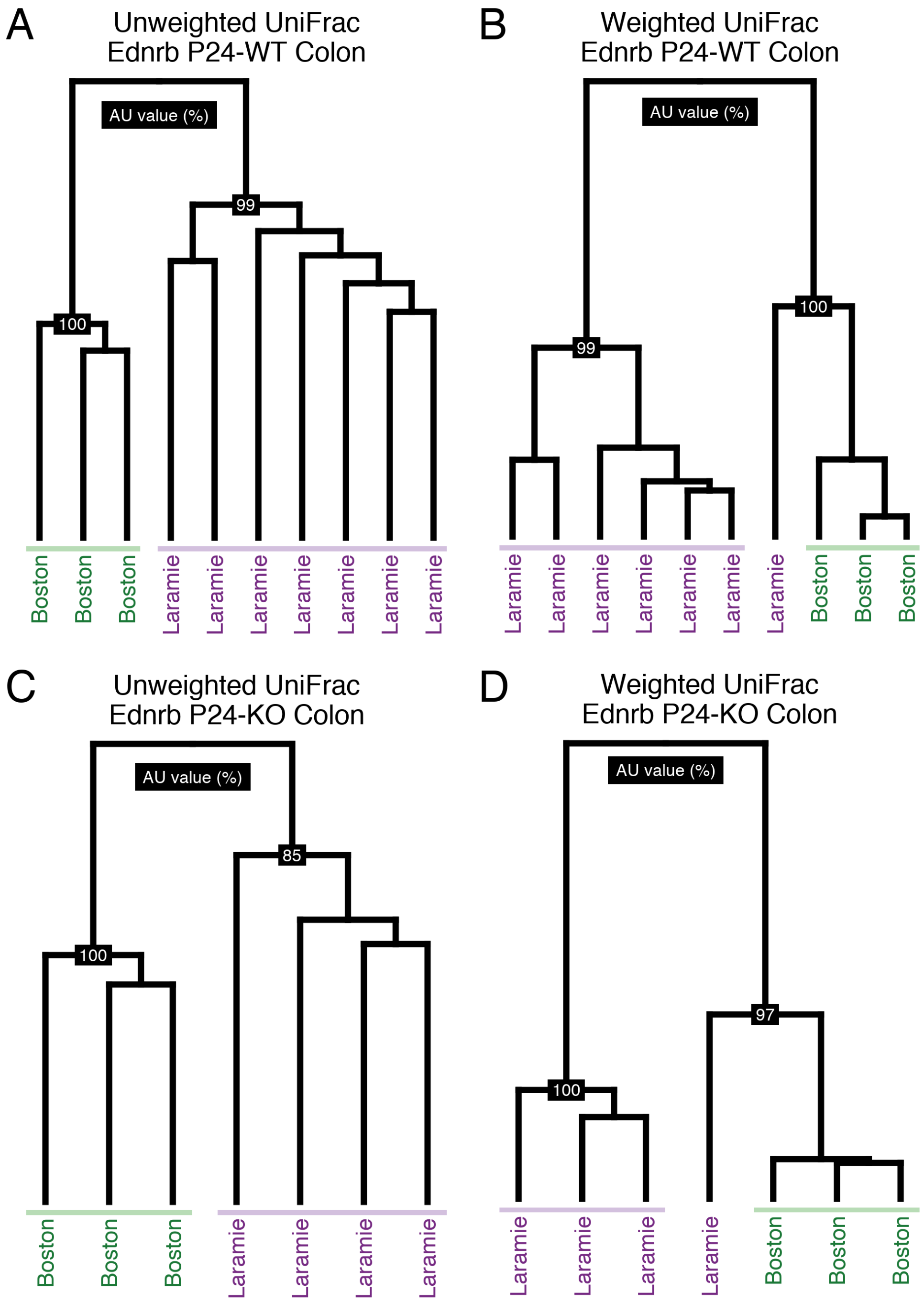

Figure S2 | Colon samples from P24 Ednrb mice cluster by facility of origin. Hierarchical clustering of unweighted (A, C) and weighted (B, D) UniFrac distances for sequences obtained from P24-WT and P24-KO colon samples. Shading emphasizes each facility's cluster.

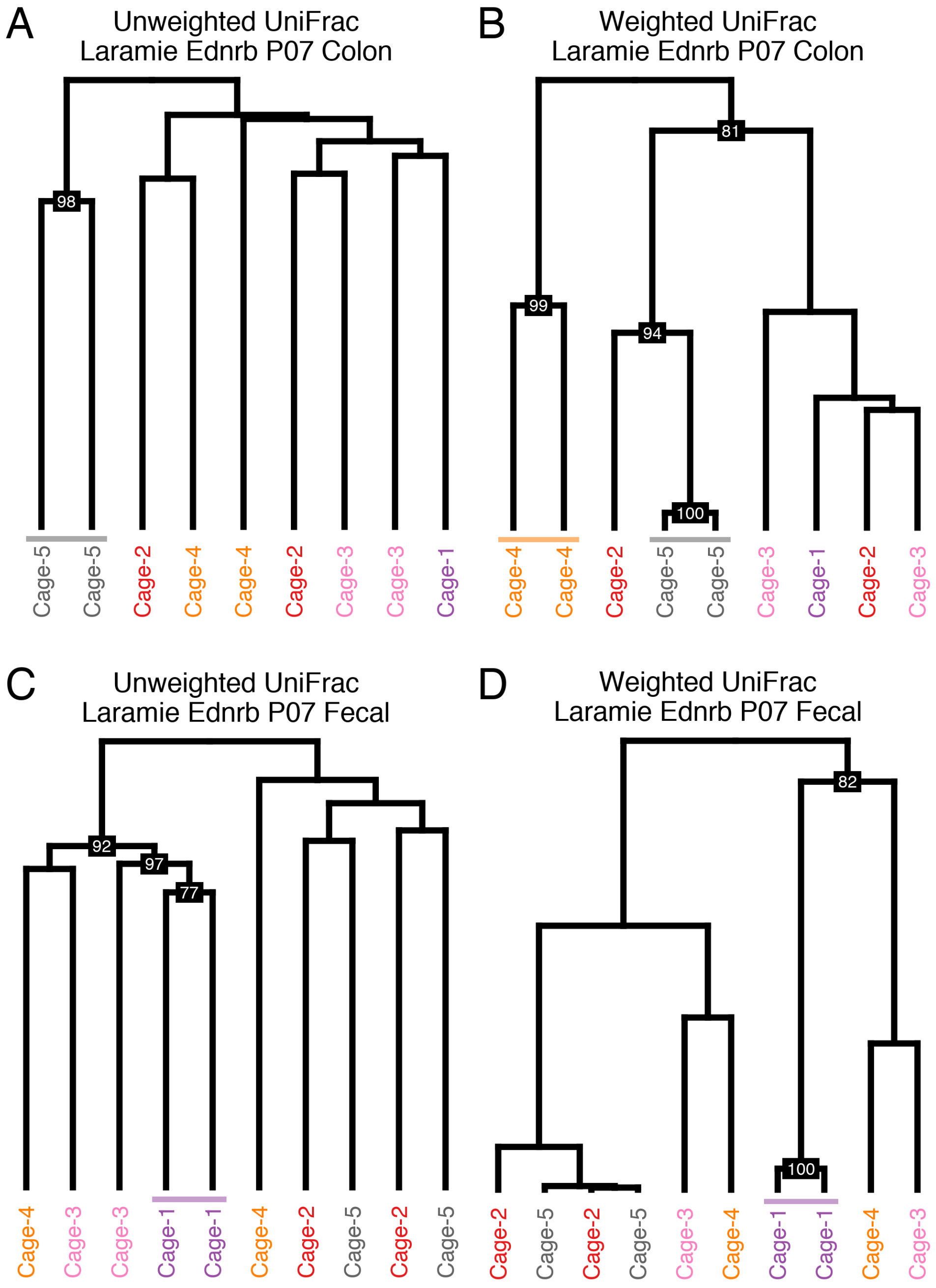

Figure S3 | Colon and fecal samples from Laramie P07 Ednrb mice exhibit some clustering by cage. Hierarchical clustering of unweighted (A, C) and weighted (B, D) UniFrac distances for sequences obtained from P07-WT and P07-KO colon and fecal samples from Laramie. Labels and colors denote separate cages. Shading represents instances where clusters contained mice from the same cage.

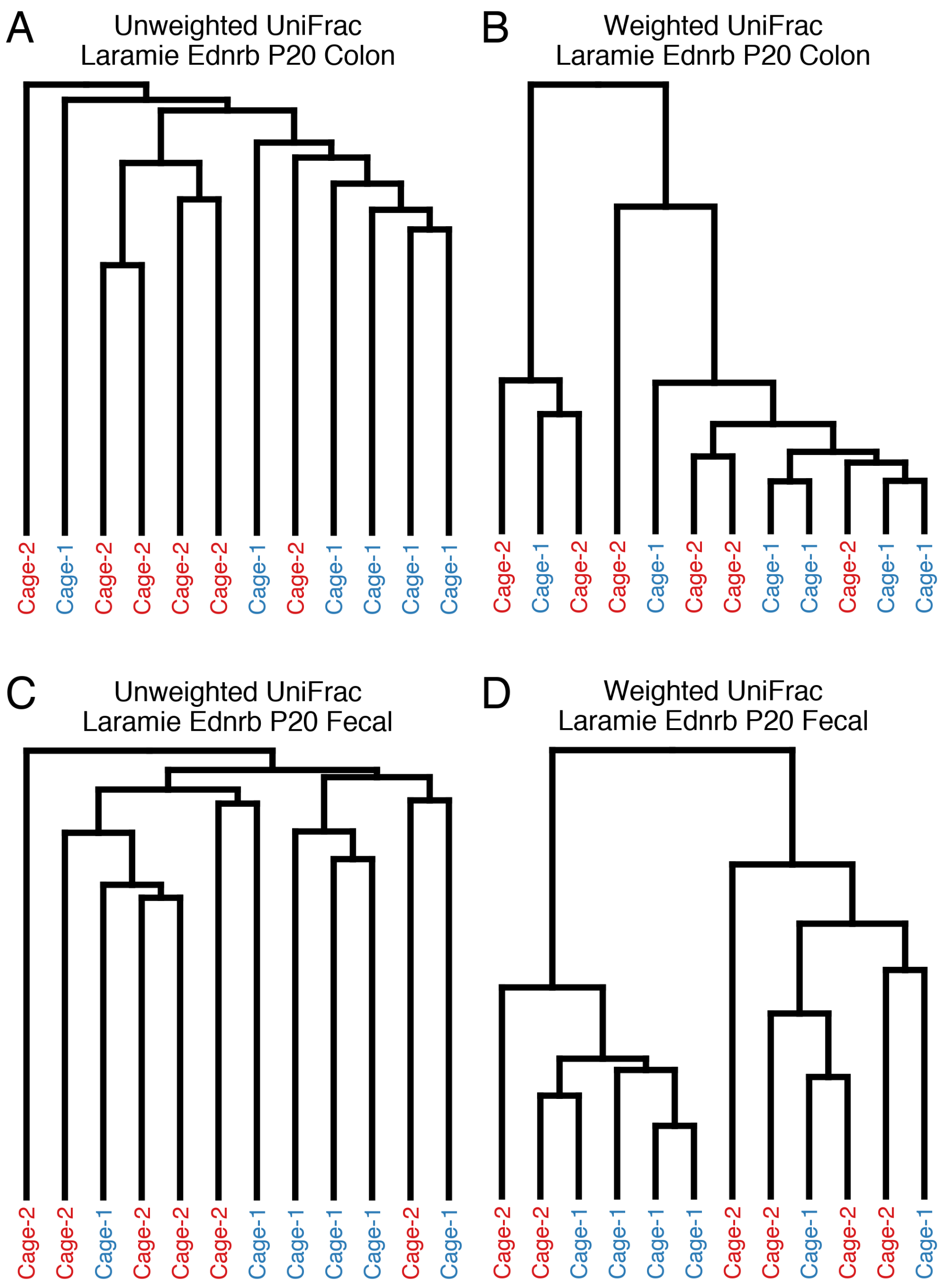

Figure S4 | Colon and fecal samples from Laramie P20 Ednrb mice exhibit no clear clustering by cage. Hierarchical clustering of unweighted (A, C) and weighted (B, D) UniFrac distances for sequences obtained from P20-WT and P20-KO colon and fecal samples from Laramie. Labels and colors denote separate cages.

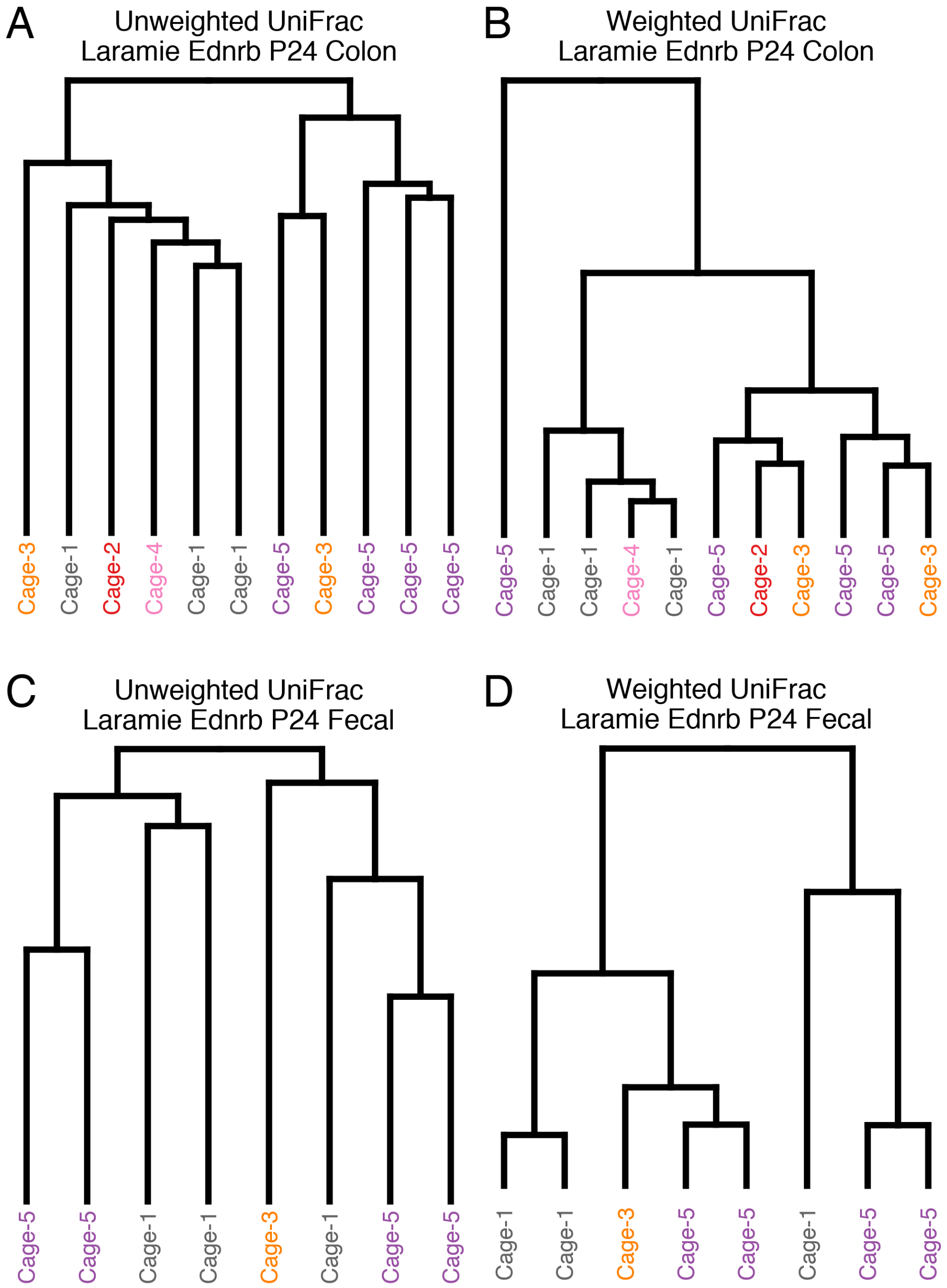

Figure S5 | Colon and fecal samples from Laramie P24 Ednrb mice exhibit no clear clustering by cage. Hierarchical clustering of unweighted (A, C) and weighted (B, D) UniFrac distances for sequences obtained from P24-WT and P24-KO colon and fecal samples from Laramie. Labels and colors denote separate cages.

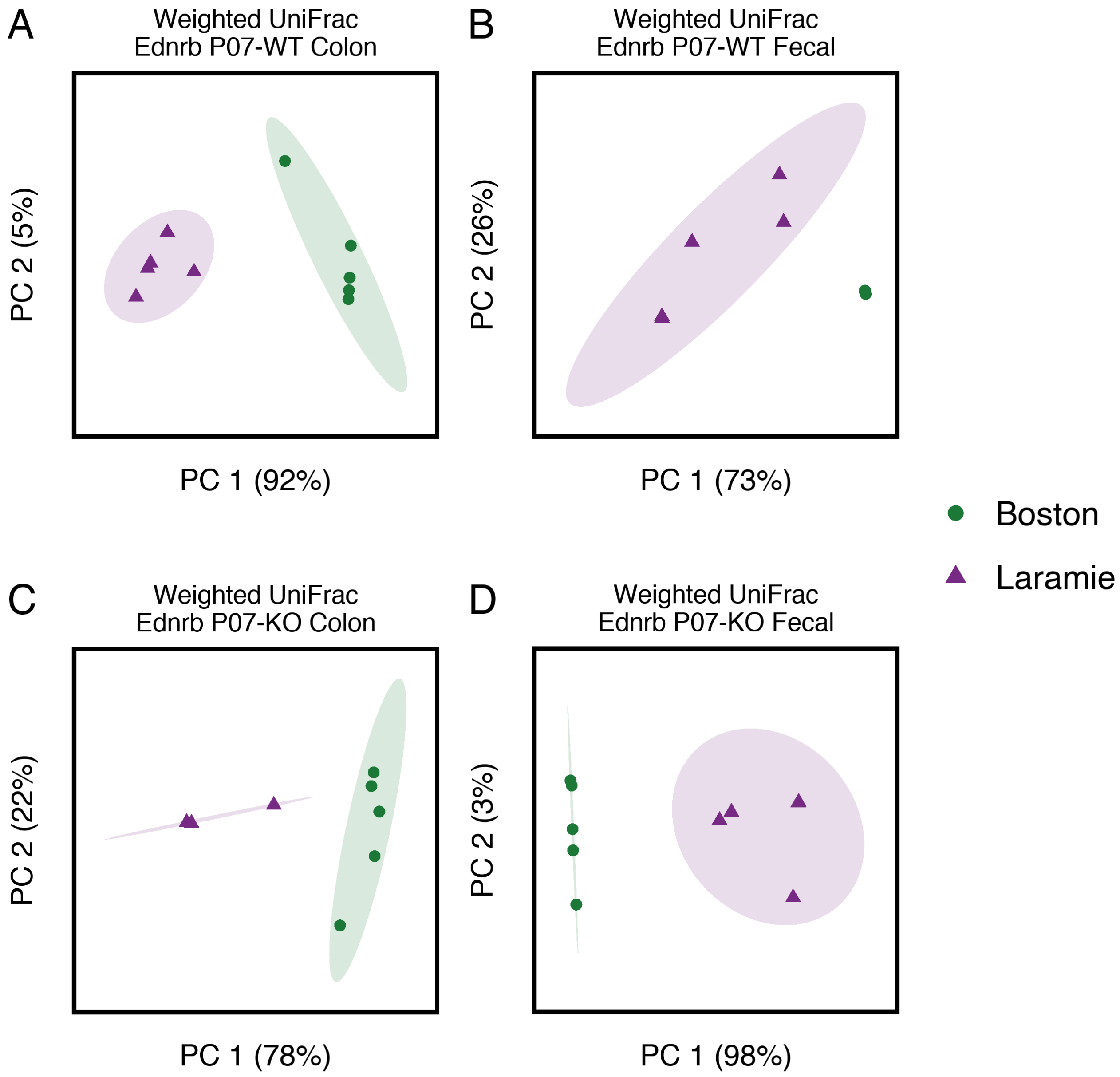

Figure S6 | Core microbiomes of P07 Ednrb mice separate by facility of origin, with no overlap. Principal Coordinates Analysis of weighted UniFrac distances for core microbiome OTUs at the 50% threshold obtained from P07-WT and P07-KO colon and fecal samples. Percentage values along each axis indicate the amount of variability in the data explained by each of the first two principal coordinates. Ellipses indicate 95% confidence intervals.

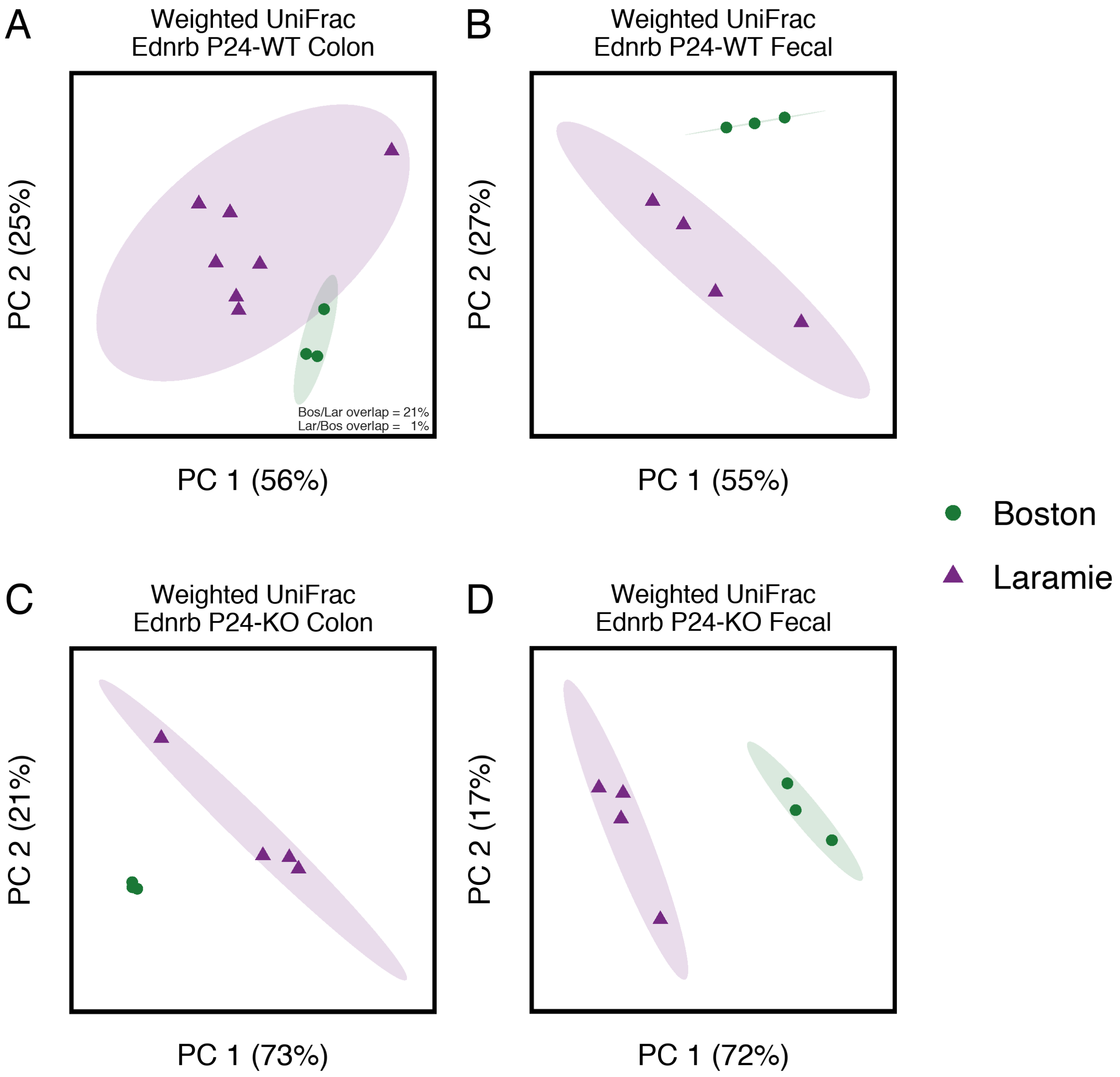

Figure S7 | Core microbiomes of P24 Ednrb mice separate by facility of origin, with very minimal overlap. Principal Coordinates Analysis of weighted UniFrac distances for core microbiome OTUs at the 50% threshold obtained from P24-WT and P24-KO colon and fecal samples. Percentage values along each axis indicate the amount of variability in the data explained by each of the first two principal coordinates. Ellipses indicate 95% confidence intervals.

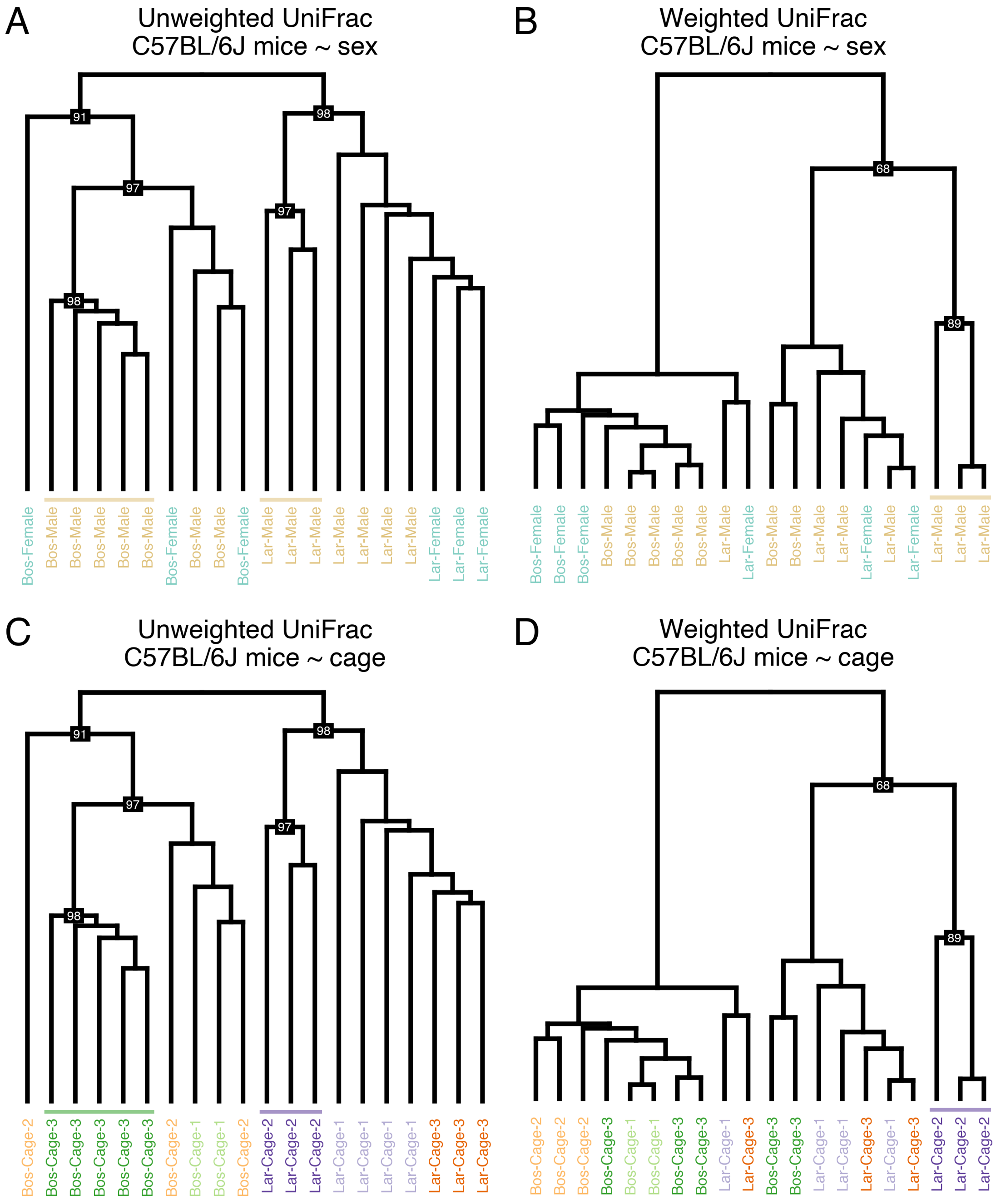

Figure S8 | Fecal samples from C57BL/6J mice show minimal clustering by sex or cage. (A-D) Hierarchical clustering of unweighted and weighted UniFrac distances for sequences obtained from C57BL/6J fecal samples. (A-B) Shading represents instances where individual clusters contained only mice of the same sex. (C-D) Each color denotes a separate cage. Shading represents instances where clusters contained only mice from the same cage.

SUPPLEMENTARY TABLES

Table S1 Inter-Facility PERMANOVA statistics

**Ednrb mice - HSCR dataset** *Boston vs. Laramie*	ADONIS								
	Total Beta Diversity						Core 50% Beta Diversity		
	Unweighted UniFrac			Weighted UniFrac			Weighted UniFrac		
Colon Samples	R2	*P*	*FDR-P*	R2	*P*	*FDR-P*	R2	*P*	*FDR-P*
P07-WT vs. P07-WT	31%	0.009	0.016	58%	0.008	0.017	86%	0.008	0.008
P20-WT vs. P20-WT	38%	0.002	0.011	78%	0.002	0.011	91%	0.002	0.002
P24-WT vs. P24-WT	29%	0.01	0.017	32%	0.03	0.037	29%	0.041	0.041
P07-KO vs. P07-KO	29%	0.007	0.016	52%	0.009	0.018	70%	0.007	0.007
P20-KO vs. P20-KO	45%	0.002	0.011	67%	0.002	0.011	73%	0.001	0.001
P24-KO vs. P24-KO	36%	0.029	0.031	54%	***0.057***	***0.064***	56%	***0.057***	***0.057***
Fecal Samples									
P07-WT vs. P07-WT	52%	0.008	0.016	63%	0.009	0.019	62%	0.009	0.009
P20-WT vs. P20-WT	33%	0.003	0.016	83%	0.003	0.015	85%	0.002	0.002
P24-WT vs. P24-WT	36%	0.029	0.03	20%	***0.343***	***0.354***	31%	***0.114***	***0.114***
P07-KO vs. P07-KO	32%	0.007	0.016	60%	0.008	0.019	92%	0.008	0.008
P20-KO vs. P20-KO	38%	0.002	0.016	74%	0.003	0.015	65%	0.003	0.003
P24-KO vs. P24-KO	43%	0.029	0.03	56%	0.029	0.034	69%	0.029	0.029
**C57BL/6J mice**									
Boston vs. Laramie	16%	0.0001	0.0001	28%	0.002	0.002	31%	0.001	0.001

Bold-Italic text indicates *FDR-P* or *P*-values > 0.05

Table S2 Core OTUs

Ednrb mice - HSCR dataset Colon Samples		Total # Observed OTUs	Core 50% # of core OTUs	Core 50% % Observed OTUs = core	Core 75% # of core OTUs	Core 75% % Observed OTUs = core	Core 100% # of core OTUs	Core 100% % Observed OTUs = core
P07-WT	Boston	20	7	35%	4	20%	3	15%
	Laramie	105	34	32%	20	19%	8	8%
	Conserved	62	14	22%	0	0%	0	0%
P07-KO	Boston	27	16	59%	7	26%	2	7%
	Laramie	67	38	57%	18	27%	11	16%
	Conserved	47	4	8%	0	0%	0	0%
P20-WT	Boston	103	42	41%	23	22%	8	8%
	Laramie	104	63	60%	23	22%	10	10%
	Conserved	104	18	17%	2	2%	0	0%
P20-KO	Boston	84	57	68%	31	37%	11	13%
	Laramie	102	57	56%	28	27%	16	16%
	Conserved	93	24	26%	4	4%	2	2%
P24-WT	Boston	150	91	61%	40	27%	40	27%
	Laramie	123	51	42%	10	8%	5	4%
	Conserved	136	50	37%	3	2%	1	1%
P24-KO	Boston	142	75	53%	23	16%	23	16%
	Laramie	93	52	56%	22	24%	6	6%
	Conserved	117	20	17%	3	3%	0	0%
**C57BL/6J mice**								
Fecal Samples	Boston	507	354	70%	163	32%	76	15%
	Laramie	528	326	62%	130	25%	65	12%
	Conserved	518	277	54%	116	22%	40	8%

Conserved is calculated as the average of Boston and Laramie. % Observed OTUs = core is calculated as the # of core OTUs divided by the Total # of Observed OTUs.

Table S3 Alpha Diversity statistics

**Ednrb mice - HSCR Dataset** *Boston vs. Laramie*	Chao1		Observed OTUs	
	Kruskal-Wallis	Pairwise Wilcoxon	Kruskal-Wallis	Pairwise Wilcoxon
Colon Samples	*FDR-P*	*FDR-P*	*FDR-P*	*FDR-P*
P07-WT vs. P07-WT	0.12	0.054	0.036**	0.07
P20-WT vs. P20-WT	0.75	0.47	0.93	0.77
P24-WT vs. P24-WT	0.36	0.12	0.48	0.45
P07-KO vs. P07-KO	0.25	0.08	0.52	0.08
P20-KO vs. P20-KO	0.025**	0.054	0.53	0.36
P24-KO vs. P24-KO	0.31	0.18	0.22	0.46
Fecal Samples				
P07-WT vs. P07-WT	0.15	0.07	0.051	0.07
P20-WT vs. P20-WT	0.54	0.24	0.87	0.88
P24-WT vs. P24-WT	0.99	1.00	0.78	1.00
P07-KO vs. P07-KO	0.54	0.08	0.50	0.07
P20-KO vs. P20-KO	0.62	0.46	0.87	0.53
P24-KO vs. P24-KO	0.59	0.14	0.39	0.12
Boston vs. Boston Colon Samples				
P07-WT vs. P07-KO	0.45	0.051	0.67	0.29
P20-WT vs. P20-KO	0.45	0.08	0.50	0.07
P24-WT vs. P24-KO	0.96	1.00	0.67	0.20
Fecal Samples				
P07-WT vs. P07-KO	0.55	0.08	0.42	0.047**
P20-WT vs. P20-KO	0.17	0.037**	0.44	0.46
P24-WT vs. P24-KO	0.60	0.41	0.37	0.11
Laramie vs. Laramie Colon Samples				
P07-WT vs. P07-KO	0.48	0.36	0.31	0.27
P20-WT vs. P20-KO	0.88	1.00	0.90	0.94
P24-WT vs. P24-KO	0.37	0.39	0.31	0.48
Fecal Samples				
P07-WT vs. P07-KO	0.57	0.62	0.51	0.74
P20-WT vs. P20-KO	0.75	0.62	0.60	0.89
P24-WT vs. P24-KO	0.97	0.89	0.60	0.89
**C57BL/6J mice**				
Boston vs. Laramie	0.26	0.27	0.45	0.47

** indicates *FDR-P*-values < 0.05

Table S4 Inter-Facility taxonomy statistics

**Ednrb mice - HSCR Dataset** Association: Facility	Group	Type	Rank	Taxon	Mean Relative Abundance	Kruskal-Wallis *FDR-P*	Pairwise Wilcoxon *FDR-P*
Boston	P07-WT	Colon	Phylum	Firmicutes	89%	0.01	NS
		Fecal	Phylum	Firmicutes	99.9%	0.03	NS
	P20-WT	Colon	Phylum	Firmicutes	73%	0.047	NS
			Family	Ruminococcaceae	6%	0.048	NS
			Genus	*Oscillospira*	9%	0.01	0.046
	P07-KO	Colon	Phylum	Firmicutes	98.7%	0.01	NS
		Fecal	Phylum	Firmicutes	98.1%	0.04	NS
	P20-KO	Colon	Family	S24-7	42%	NS	0.04
		Fecal	Order	Clostridiales	17%	NS	0.047
			Family	S24-7	60%	0.04	0.04
Laramie	P07-WT	Colon	Phylum	Actinobacteria	19%	0.01	NS
			Phylum	Proteobacteria	46%	0.02	NS
			Family	Enterobacteriaceae	35%	0.01	NS
			Genus	*Propionibacterium*	11%	0.002	0.04
		Fecal	Phylum	Actinobacteria	9%	0.002	0.046
			Phylum	Proteobacteria	50%	0.001	NS
			Family	Enterobacteriaceae	44%	0.002	0.04
	P20-WT	Colon	Order	Bacteroidales	12%	0.00	0.02
			Genus	*Bacteroides*	25%	0.01	0.02
			Genus	*Parabacteroides*	9%	0.01	0.02
		Fecal	Phylum	Bacteroidetes	79%	NS	0.03
			Order	Bacteroidales-1	11%	0.003	0.03
			Order	Bacteroidales-2	7%	0.003	0.03
			Genus	*Bacteroides*	32%	0.004	0.03
			Genus	*Parabacteroides*	8%	0.004	0.03
	P24-WT	Colon	Genus	*Bacteroides*	20%	0.02	0.04
		Fecal	Order	Bacteroidales	9%	0.04	NS
	P07-KO	Colon	Phylum	Actinobacteria	7%	0.02	NS
			Phylum	Proteobacteria	58%	0.02	NS
			Family	Enterobacteriaceae	54%	0.01	NS
		Fecal	Phylum	Proteobacteria	55%	0.02	NS
			Family	Enterobacteriaceae	54%	0.02	0.047
	P20-KO	Colon	Phylum	Bacteroidetes	77%	NS	0.04
			Order	Bacteroidales-1	14%	0.003	0.02
			Order	Bacteroidales-2	8%	0.003	0.02
			Genus	*Bacteroides*	34%	0.01	0.02
			Genus	*Parabacteroides*	7%	0.01	0.02
		Fecal	Phylum	Bacteroidetes	85%	NS	0.03
			Order	Bacteroidales-1	12%	0.003	0.03
			Order	Bacteroidales-2	7%	0.003	0.03
			Genus	*Bacteroides*	48%	0.001	0.03
			Genus	*Parabacteroides*	7%	0.002	0.03
	P24-KO	Colon	Genus	*Bacteroides*	37%	0.01	NS
			Genus	*Parabacteroides*	14%	0.02	NS
		Fecal	Genus	*Bacteroides*	39%	0.02	NS
			Genus	*Parabacteroides*	22%	0.01	NS
**C57BL/6J mice**							
Boston		Fecal	Phylum	Bacteroidetes	76%	0.03	0.03
Laramie		Fecal	Phylum	Firmicutes	39%	0.01	0.01

Only those taxa with mean relative abundances above 6% and that exhibited significant differences between age- and genotype-matched Boston and Laramie mice are shown.

Table S5 Intra-Facility PERMANOVA statistics

**Ednrb mice - HSCR dataset**	ADONIS					
	Total Beta Diversity					
	Unweighted UniFrac			Weighted UniFrac		
Boston vs. Boston						
Colon Samples	R2	*P*	*FDR-P*	R2	*P*	*FDR-P*
P07-WT vs. P07-KO	14%	0.084	0.090	35%	0.074	0.079
P20-WT vs. P20-KO	28%	0.008**	0.022**	68%	0.007**	0.026**
P24-WT vs. P24-KO	24%	0.100	0.100	48%	0.100	0.100
Fecal Samples						
P07-WT vs. P07-KO	44%	0.007**	0.02**	19%	0.096	0.100
P20-WT vs. P20-KO	25%	0.007**	0.02**	80%	0.008**	0.022**
P24-WT vs. P24-KO	30%	0.100	0.100	34%	0.100	0.100
Laramie vs. Laramie						
Colon Samples						
P07-WT vs. P07-KO	14%	0.212	0.212	17%	0.220	0.253
P20-WT vs. P20-KO	11%	0.064	0.069	6%	0.620	0.620
P24-WT vs. P24-KO	15%	0.027**	0.032**	15%	0.141	0.176
Fecal Samples						
P07-WT vs. P07-KO	12%	0.315	0.315	12%	0.404	0.433
P20-WT vs. P20-KO	11%	0.102	0.109	8%	0.507	0.507
P24-WT vs. P24-KO	24%	0.028**	0.035**	23%	0.200	0.231

** indicates *FDR-P* or *P*-values < 0.05

Table S6 Intra-Facility taxonomy statistics

**Ednrb mice - HSCR Dataset** Association: Facility	Type	Group	Rank	Taxon	Mean Relative Abundance	Kruskal-Wallis *FDR-P*	Pairwise Wilcoxon *FDR-P*
Boston	Colon	P20-KO	Family	Enterobacteriaceae	7%	0.03	NS
	Fecal	P07-WT	Phylum	Firmicutes	99.9%	NS	0.04
		P20-KO	Phylum	Bacteroidetes	60%	NS	0.04
			Family	S24-7	60%	NS	0.04
Laramie	Fecal	P20-KO	Phylum	Proteobacteria	6%	NS	0.03
			Family	Enterobacteriaceae	6%	NS	0.04
		P24-KO	Family	Enterobacteriaceae	11%	NS	0.049
			Genus	*Bacteroides*	39%	NS	0.046
			Genus	*Parabacteroides*	22%	0.03	NS
		P20-WT	Family	S24-7	20%	NS	0.04
		P24-WT	Family	S24-7	26%	NS	0.04

Only those taxa with mean relative abundances above 6% and that exhibited significant differences between age-matched Boston or Laramie mice are shown.

Table S7 Differentially abundant non-candidate OTUs and taxa conserved between facilities

Ednrb mice - HSCR Dataset

OTU-ID	Taxon	Statistical Test	Type	Facility	Association	Log_2 FC
549756^	G: *Lactobacillus*	G-Test	Fecal	Boston	P07-WT	-0.29
				Laramie	P07-KO	+2.4
n/a	G: *Streptococcus*	Kruskal-Wallis	Fecal	Boston	P07-KO	+10
				Laramie	P07-WT	-2.5
n/a	P: Actinobacteria	Kruskal-Wallis	Colon	Boston	P20-KO	+10
				Laramie	P20-WT	-6.7
		Nonparametric T		Boston	P20-KO	+10
				Laramie	P20-WT	-6.7
n/a	F: S24-7^	Kruskal-Wallis	Fecal	Boston	P20-KO	+1.4
				Laramie	P20-WT	-0.94
		Nonparametric T		Boston	P20-KO	+1.4
				Laramie	P20-WT	-0.94
n/a	F: Ruminococcaceae	Kruskal-Wallis	Fecal	Boston	P24-KO	+2.7
				Laramie	P24-WT	-4.8

^ Indicates OTUs or taxa observed at multiple ages. P: represents Phylum. F: represents Family. G: represents Genus. Log_2 Fold Change (FC) was calculated KO/WT, therefore (+) indicates association with KO mice and (-) indicates association with WT mice.

Table S8 Rarefaction depths

OTU Table Name	Depth
HSCR_OTU_table	374
cBL_HSCR	374
fBL_HSCR	396
cBos_HSCR	695
fBos_HSCR	1791
cLar_HSCR	374
fLar_HSCR	396
C57_OTU_table	12790

DATA SHEET 2 FILE KEY

-QIIME_Code_1 (.html) Command-line code for demultiplexing, quality filtering, and OTU picking using QIIME 1.
-QIIME_Code_2 (.html) Command-line code for OTU table filtering, rarefaction, and all analyses using QIIME 1.
-Pre_R_Code.html (.html) Command-line code for moving QIIME 1 outputs prior to using R.
-HSCR_metadata (.txt) Tab-delimited text file of metadata containing information about all samples from the HSCR studies.
-HSCR_OTU_table (.biom) BIOM formatted file of unrarefied OTU counts of observations and assigned taxonomies for all samples from the HSCR studies.
-HSCR_rep_set (.tre) FastTree formatted file containing phylogenetic tree of representative sequences for all samples from the HSCR studies.
-HSCR_diffabund_map (.txt) Tab-delimited text file of metadata formatted for differential abundance testing (using QIIME 1) of samples from the HSCR studies.
-C57_metadata (.txt) Tab-delimited text file of metadata containing information about all samples from the C57BL/6J study.
-C57_OTU_table (.biom) BIOM formatted file of unrarefied OTU counts of observations and assigned taxonomies for all samples from the C57BL/6J study.
-C57_rep_set (.tre) FastTree formatted file containing phylogenetic tree of representative sequences for all samples from the C57BL/6J study.
-hscr_R_meta (.txt) Tab-delimited text file (R map) for the HSCR studies, needed when working in R.
-hscr_R_format_file (.txt) Tab-delimited text file (R format file) for the HSCR studies, needed when working in R.
-c57_R_ meta (.txt) Tab-delimited text file (R map) for the C57BL/6J study, needed when working in R.
-c57_R_format_file (.txt) Tab-delimited text file (R format file) for the C57BL/6J study, needed when working in R.
-R_Code_1 (.R) R code for dendrogram construction and visualization to generate Figure 1, Supplementary Figures 1-5, and for principal coordinates analysis and visualization to generate Figure 2, and Supplementary Figures 6 and 7.
-R_Code_1_WS (.Rdata) The final R workspace generated for Figure 1-2, and Supplementary Figures 1-7.
-R_Code_2 (.R) R code for generating Figure 3.
-R_Code_2_WS (.Rdata) The final R workspace generated for Figure 3.
-R_Code_3 (.R) R code for generating Figure 4 and Supplementary Figure 8.
-R_Code_3_WS (.Rdata) The final R workspace generated for Figure 4 and Supplementary Figure 8.
-R_Code_4 (.R) R code for generating values for Table 1, and Supplementary Tables 1, 2, 3, 4, 5, 6, and 7.
-R_Code_4_WS (.Rdata) The final R workspace generated for Table 1, and Supplementary Tables 1, 2, 3, 4, 5, 6, and 7.
