# Supplementary material for: Microbiome Composition in Both Wild-Type and Disease Model Mice Is Heavily Influenced by Mouse Facility
Source: Front Microbiol. 2018 Jul 20;9:1598. doi: 10.3389/fmicb.2018.01598 (PMC6062620; doi:10.3389/fmicb.2018.01598)
Supplement: Supplementary file 2 [file Data_Sheet_2.ZIP › DataSheet2/Pre_R_Code.html]

Pre\_R\_Code


## Command-line code for moving QIIME 1 outputs prior to using R Studio.¶

### Setup directories and files - from completing QIIME\_Code\_2.html¶

In order to run in "copy-paste mode," you will need to have setup the directory hierarchy found in the the file QIIME\_Code\_2.html and performed the commands in QIIME\_Code\_2.html. Otherwise, what you need for each Figure or Table is listed under its respective heading.

In [ ]:

```
# change working directory
cd Desktop/

# make new directories
mkdir r_analysis/
mkdir r_analysis/bdiv/
mkdir r_analysis/fig_1/
mkdir r_analysis/fig_s1/
mkdir r_analysis/fig_s2/
mkdir r_analysis/tab_s1/
mkdir r_analysis/fig_s3/
mkdir r_analysis/fig_s4/
mkdir r_analysis/fig_s5/
mkdir r_analysis/fig_2/
mkdir r_analysis/fig_s6/
mkdir r_analysis/fig_s7/
mkdir r_analysis/fig_3/
mkdir r_analysis/fig_4/
mkdir r_analysis/fig_s8/
mkdir r_analysis/tab_1_tab_s7/
mkdir r_analysis/tab_s2/
mkdir r_analysis/tab_s3/
mkdir r_analysis/tab_s4_tab_s6/
mkdir r_analysis/tab_s5/
```

### Setup metadata, format files, and .R files - from downloading DataSheet2.zip¶

Download the files in DataSheet2.zip. From wherever files are downloaded, copy them to the appropriate locations, specified below.

In [ ]:

```
# move files (in this case, they were located in "~/Downloads")
# change working directory
cd ../Downloads/

# copy the metadata, format, and .R files
cp DataSheet2/hscr_R_meta.txt ../Desktop/r_analysis/hscr_R_meta.txt
cp DataSheet2/hscr_R_format_file.txt ../Desktop/r_analysis/hscr_R_format_file.txt
cp DataSheet2/c57_R_meta.txt ../Desktop/r_analysis/c57_R_meta.txt
cp DataSheet2/c57_R_format_file.txt ../Desktop/r_analysis/c57_R_format_file.txt
cp DataSheet2/R_Code_1.R ../Desktop/r_analysis/R_Code_1.R
cp DataSheet2/R_Code_2.R ../Desktop/r_analysis/R_Code_2.R
cp DataSheet2/R_Code_3.R ../Desktop/r_analysis/R_Code_3.R
cp DataSheet2/R_Code_4.R ../Desktop/r_analysis/R_Code_4.R

# change working directory back to Desktop
cd ../Desktop/
```

### Figure 1 - Figure S1 - Figure S2¶

File locations:

- q1\_analysis/hscr/bdiv/unweighted\_unifrac\_cBL\_HSCR\_rare.txt
- q1\_analysis/hscr/bdiv/weighted\_unifrac\_cBL\_HSCR\_rare.txt

We will filter the distance matrices for the appropriate groups in R.

In [ ]:

```
# copy files
cp q1_analysis/hscr/bdiv/unweighted_unifrac_cBL_HSCR_rare.txt r_analysis/bdiv/uw_cBL.txt
cp q1_analysis/hscr/bdiv/weighted_unifrac_cBL_HSCR_rare.txt r_analysis/bdiv/w_cBL.txt
```

### Figure S3 - Figure S4 - Figure S5¶

File locations:

- q1\_analysis/hscr/bdiv/unweighted\_unifrac\_cLar\_HSCR\_rare.txt
- q1\_analysis/hscr/bdiv/weighted\_unifrac\_cLar\_HSCR\_rare.txt
- q1\_analysis/hscr/bdiv/unweighted\_unifrac\_fLar\_HSCR\_rare.txt
- q1\_analysis/hscr/bdiv/weighted\_unifrac\_fLar\_HSCR\_rare.txt

We will filter the distance matrices for the appropriate groups in R.

In [ ]:

```
# copy files
cp q1_analysis/hscr/bdiv/unweighted_unifrac_cLar_HSCR_rare.txt r_analysis/bdiv/uw_cLar.txt
cp q1_analysis/hscr/bdiv/weighted_unifrac_cLar_HSCR_rare.txt r_analysis/bdiv/w_cLar.txt
cp q1_analysis/hscr/bdiv/unweighted_unifrac_cLar_HSCR_rare.txt r_analysis/bdiv/uw_fLar.txt
cp q1_analysis/hscr/bdiv/weighted_unifrac_cLar_HSCR_rare.txt r_analysis/bdiv/w_fLar.txt
```

### Figure 2¶

File locations:

- q1\_analysis/hscr/core\_bdiv/weighted\_unifrac\_cBL\_P20WTcore50.txt
- q1\_analysis/hscr/core\_bdiv/weighted\_unifrac\_fBL\_P20WTcore50.txt
- q1\_analysis/hscr/core\_bdiv/weighted\_unifrac\_cBL\_P20KOcore50.txt
- q1\_analysis/hscr/core\_bdiv/weighted\_unifrac\_fBL\_P20KOcore50.txt

In [ ]:

```
# copy files
cp q1_analysis/hscr/core_bdiv/weighted_unifrac_cBL_P20WTcore50.txt r_analysis/fig_2/w_cBL_P20WTcore.txt
cp q1_analysis/hscr/core_bdiv/weighted_unifrac_fBL_P20WTcore50.txt r_analysis/fig_2/w_fBL_P20WTcore.txt
cp q1_analysis/hscr/core_bdiv/weighted_unifrac_cBL_P20KOcore50.txt r_analysis/fig_2/w_cBL_P20KOcore.txt
cp q1_analysis/hscr/core_bdiv/weighted_unifrac_fBL_P20KOcore50.txt r_analysis/fig_2/w_fBL_P20KOcore.txt
```

### Figure S6¶

File locations:

- q1\_analysis/hscr/core\_bdiv/weighted\_unifrac\_cBL\_P07WTcore50.txt
- q1\_analysis/hscr/core\_bdiv/weighted\_unifrac\_fBL\_P07WTcore50.txt
- q1\_analysis/hscr/core\_bdiv/weighted\_unifrac\_cBL\_P07KOcore50.txt
- q1\_analysis/hscr/core\_bdiv/weighted\_unifrac\_fBL\_P07KOcore50.txt

In [ ]:

```
# copy files
cp q1_analysis/hscr/core_bdiv/weighted_unifrac_cBL_P07WTcore50.txt r_analysis/fig_s6/w_cBL_P07WTcore.txt
cp q1_analysis/hscr/core_bdiv/weighted_unifrac_fBL_P07WTcore50.txt r_analysis/fig_s6/w_fBL_P07WTcore.txt
cp q1_analysis/hscr/core_bdiv/weighted_unifrac_cBL_P07KOcore50.txt r_analysis/fig_s6/w_cBL_P07KOcore.txt
cp q1_analysis/hscr/core_bdiv/weighted_unifrac_fBL_P07KOcore50.txt r_analysis/fig_s6/w_fBL_P07KOcore.txt
```

### Figure S7¶

File locations:

- q1\_analysis/hscr/core\_bdiv/weighted\_unifrac\_cBL\_P24WTcore50.txt
- q1\_analysis/hscr/core\_bdiv/weighted\_unifrac\_fBL\_P24WTcore50.txt
- q1\_analysis/hscr/core\_bdiv/weighted\_unifrac\_cBL\_P24KOcore50.txt
- q1\_analysis/hscr/core\_bdiv/weighted\_unifrac\_fBL\_P24KOcore50.txt

In [ ]:

```
# copy files
cp q1_analysis/hscr/core_bdiv/weighted_unifrac_cBL_P24WTcore50.txt r_analysis/fig_s7/w_cBL_P24WTcore.txt
cp q1_analysis/hscr/core_bdiv/weighted_unifrac_fBL_P24WTcore50.txt r_analysis/fig_s7/w_fBL_P24WTcore.txt
cp q1_analysis/hscr/core_bdiv/weighted_unifrac_cBL_P24KOcore50.txt r_analysis/fig_s7/w_cBL_P24KOcore.txt
cp q1_analysis/hscr/core_bdiv/weighted_unifrac_fBL_P24KOcore50.txt r_analysis/fig_s7/w_fBL_P24KOcore.txt
```

### Figure 3¶

File location:

- q1\_analysis/hscr/taxa/HSCR\_phylum.txt

In [ ]:

```
# copy file
cp q1_analysis/hscr/taxa/HSCR_phylum.txt r_analysis/fig_3/HSCR_phylum.txt
```

### Figure 4 - Figure S8¶

File locations:

- q1\_analysis/c57/bdiv/unweighted\_unifrac\_C57\_OTU\_table\_rare.txt
- q1\_analysis/c57/bdiv/weighted\_unifrac\_C57\_OTU\_table\_rare.txt
- q1\_analysis/c57/core\_bdiv/weighted\_unifrac\_c57core50.txt
- q1\_analysis/c57/taxa/c57\_phylum.txt

In [ ]:

```
# copy files
cp q1_analysis/c57/bdiv/unweighted_unifrac_C57_OTU_table_rare.txt r_analysis/bdiv/uw_c57.txt
cp q1_analysis/c57/bdiv/weighted_unifrac_C57_OTU_table_rare.txt r_analysis/bdiv/w_c57.txt
cp q1_analysis/c57/core_bdiv/weighted_unifrac_c57core50.txt r_analysis/fig_4/c57core50.txt
cp q1_analysis/c57/taxa/c57_phylum.txt r_analysis/fig_4/c57_phylum.txt
```

### Table 1 - Table S7¶

Directory locations:

- q1\_analysis/hscr/diff\_abund/otus/
- q1\_analysis/hscr/diff\_abund/taxa/

**NOTE:** copy the directories of interest

In [ ]:

```
# copy directories
cp -r q1_analysis/hscr/diff_abund/otus/ r_analysis/tab_1_tab_s7/otus/
cp -r q1_analysis/hscr/diff_abund/taxa/ r_analysis/tab_1_tab_s7/taxa/
```

### Table S1¶

File locations:

- q1\_analysis/hscr/bdiv/unweighted\_unifrac\_fBL\_HSCR\_rare.txt
- q1\_analysis/hscr/bdiv/weighted\_unifrac\_fBL\_HSCR\_rare.txt

We will filter the distance matrices for the appropriate groups in R.

**NOTE:** cBL files were moved for Figure 1, S1, S2 and C57 files were moved for Figure 4, S8

In [ ]:

```
# copy files
cp q1_analysis/hscr/bdiv/unweighted_unifrac_fBL_HSCR_rare.txt r_analysis/bdiv/uw_fBL.txt
cp q1_analysis/hscr/bdiv/weighted_unifrac_fBL_HSCR_rare.txt r_analysis/bdiv/w_fBL.txt
```

### Table S2¶

File locations:

- q1\_analysis/hscr/adiv/inter/cBL\_HSCR\_adiv.txt
- q1\_analysis/c57/adiv/c57\_adiv.txt

Directory location:

- q1\_analysis/hscr/core\_comp/

- q1\_analysis/c57/core\_comp/
**NOTE:** copy the directories of interest

In [ ]:

```
# copy files
cp q1_analysis/hscr/adiv/inter/cBL_HSCR_adiv.txt r_analysis/tab_s2/cBL_HSCR_adiv.txt
cp q1_analysis/c57/adiv/c57_adiv.txt r_analysis/tab_s2/c57_adiv.txt
cp -r q1_analysis/hscr/core_comp/ r_analysis/tab_s2/hscr_core_comp/
cp -r q1_analysis/c57/core_comp/ r_analysis/tab_s2/c57_core_comp/
```

### Table S3¶

Directory locations:

- q1\_analysis/hscr/adiv/
- q1\_analysis/c57/adiv/

**NOTE:** copy the directories of interest

In [ ]:

```
# copy directories
cp -r q1_analysis/hscr/adiv/ r_analysis/tab_s3/hscr/
cp -r q1_analysis/c57/adiv/ r_analysis/tab_s3/c57/
```

### Table S4 - Table S6¶

File locations:

- q1\_analysis/hscr/taxa/HSCR\_phylum.txt
- q1\_analysis/hscr/taxa/HSCR\_genus.txt
- q1\_analysis/c57/taxa/c57\_phylum.txt

In [ ]:

```
# copy files
cp q1_analysis/hscr/taxa/HSCR_phylum.txt r_analysis/tab_s4_tab_s6/HSCR_phylum.txt
cp q1_analysis/hscr/taxa/HSCR_genus.txt r_analysis/tab_s4_tab_s6/HSCR_genus.txt
cp q1_analysis/c57/taxa/c57_phylum.txt r_analysis/tab_s4_tab_s6/c57_phylum.txt
```

### Table S5¶

File locations:

- q1\_analysis/hscr/bdiv/unweighted\_unifrac\_cBos\_HSCR\_rare.txt
- q1\_analysis/hscr/bdiv/weighted\_unifrac\_cBos\_HSCR\_rare.txt
- q1\_analysis/hscr/bdiv/unweighted\_unifrac\_fBos\_HSCR\_rare.txt
- q1\_analysis/hscr/bdiv/weighted\_unifrac\_fBos\_HSCR\_rare.txt

We will filter the distance matrices for the appropriate groups in R.

**NOTE:** cLar and fLar files were moved for Figure S3, S4, S5

In [ ]:

```
# copy files
cp q1_analysis/hscr/bdiv/unweighted_unifrac_cBos_HSCR_rare.txt r_analysis/bdiv/uw_cBos.txt
cp q1_analysis/hscr/bdiv/weighted_unifrac_cBos_HSCR_rare.txt r_analysis/bdiv/w_cBos.txt
cp q1_analysis/hscr/bdiv/unweighted_unifrac_fBos_HSCR_rare.txt r_analysis/bdiv/uw_fBos.txt
cp q1_analysis/hscr/bdiv/weighted_unifrac_fBos_HSCR_rare.txt r_analysis/bdiv/w_fBos.txt
```

##### Achievement unlocked. Proceed to your choice of .R files for generating figures or table in R Studio¶
