# Supplementary material for: Microbiome Composition in Both Wild-Type and Disease Model Mice Is Heavily Influenced by Mouse Facility
Source: Front Microbiol. 2018 Jul 20;9:1598. doi: 10.3389/fmicb.2018.01598 (PMC6062620; doi:10.3389/fmicb.2018.01598)
Supplement: Supplementary file 2 [file Data_Sheet_2.ZIP › DataSheet2/QIIME_Code_1.html]

QIIME\_Code\_1


## Command-line code for demultiplexing, quality filtering, and OTU picking using QIIME 1.¶

This file includes:

- Demultiplexing
- Quality filtering
- OTU picking

### Setup directory hierarchy¶

In order to run in copy-paste mode you will need to setup a directory hierarchy using the commands below.

In [ ]:

```
# change working directory
cd Desktop/

# make new directories
mkdir q1_analysis/
mkdir q1_analysis/hscr/
mkdir q1_analysis/hscr/fna_map_qual/
mkdir q1_analysis/c57/
mkdir q1_analysis/params/
```

### Setup files¶

You will also need to move the appropriate files to the appropriate locations. I cannot be sure where you downloaded the files from, so you will have to do your best to get everything in the right locations. Here is what you will need for the commands found below.

File locations:

- q1\_analysis/HSCR\_metadata.txt
- q1\_analysis/C57\_metadata.txt
- q1\_analysis/HSCR\_diffabund\_map.txt
- q1\_analysis/hscr/fna\_map\_qual/hscr\_map454.txt
- q1\_analysis/hscr/fna\_map\_qual/hscr\_Boston\_qual.qual
- q1\_analysis/hscr/fna\_map\_qual/hscr\_Boston\_seqs.fna
- q1\_analysis/hscr/fna\_map\_qual/hscr\_Laramie454\_qual.qual
- q1\_analysis/hscr/fna\_map\_qual/hscr\_Laramie454\_seqs.fna
- q1\_analysis/hscr/fna\_map\_qual/hscr\_LaramieIllum\_map.txt
- q1\_analysis/hscr/fna\_map\_qual/hscr\_LaramieIllum\_qual.qual
- q1\_analysis/hscr/fna\_map\_qual/hscr\_LaramieIllum\_seqs.fna
- q1\_analysis/c57/C57BL6J\_seqs.fna
- q1\_analysis/params/OTUpick\_params.txt

### Activate the QIIME 1 environment and change working directory¶

In [ ]:

```
# version: 1.9.1
source activate qiime1

# change working directory
cd Desktop/q1_analysis/
```

### Validate mapping files¶

If errors or warnings are found, something is wrong with the downloaded files. Try downloading them again.

In [ ]:

```
validate_mapping_file.py -m hscr/fna_map_qual/hscr_map454.txt -o hscr/fna_map_qual/map_val_454/
validate_mapping_file.py -m hscr/fna_map_qual/hscr_LaramieIllum_map.txt -o hscr/fna_map_qual/map_val_LarIllum/
```

### Demultiplex and quality filter the sequence files¶

The 454 runs will be demultiplexed in the same line. For the illumina sequence file *hscr\_LaramieIllum\_seqs.fna*, the -p flag tells the command to ignore the primer. The Illumina data obtained from our sequencing facility (RTL Genomics) does not contain the primer.

**NOTE:** The sequence file from the C57BL/6J dataset, *C57BL6J\_seqs.fna*, was filtered from a larger dataset after demultiplexing using identical parameters as below.

In [ ]:

```
# HSCR dataset - 454
split_libraries.py -m hscr/fna_map_qual/hscr_map454.txt -f hscr/fna_map_qual/hscr_Boston_seqs.fna,hscr/fna_map_qual/hscr_Laramie454_seqs.fna -q hscr/fna_map_qual/hscr_Boston_qual.qual,hscr/fna_map_qual/hscr_Laramie454_qual.qual -b 8 -o hscr/454splt_lib/ 

# HSCR dataset - illumina
split_libraries.py -m hscr/fna_map_qual/hscr_LaramieIllum_map.txt -f hscr/fna_map_qual/hscr_LaramieIllum_seqs.fna -q hscr/fna_map_qual/hscr_LaramieIllum_qual.qual -p -b 8 -o hscr/illsplt_lib/
```

### Check sequence statistics¶

To ensure that demultiplexing and quality filtering proceeded properly, obtain sequence counts for the split\_libraries.py outputs.

In [ ]:

```
# HSCR dataset
# 454 seqs
count_seqs.py -i hscr/454_splt_lib/seqs.fna -o hscr/454_splt_lib/454_count.txt

# Laramie illumina seqs
count_seqs.py -i hscr/illsplt_lib/seqs.fna -o hscr/illsplt_lib/ill_count.txt

# C57BL/6J dataset
count_seqs.py -i c57/C57BL6J_seqs.fna -o c57/c57_count.txt
```

#### Summary of sequence statistics¶

- *454\_count.txt* = **734,859** averaging **397bp** in length
- *ill\_count.txt* = **247,198** averaging **320bp** in length
- *c57\_count.txt* = **700,191** averaging **321p** in length

### Merge HSCR 454 and Laramie Illumina sequences and check sequence statistics¶

Prior to OTU pucking, merge the HSCR 454 and Laramie Illumina sequences and check sequence statistics to ensure that the files merged properly.

In [ ]:

```
# merge the sequence files using cat
cat hscr/454splt_lib/seqs.fna hscr/illsplt_lib/seqs.fna > hscr/seqs_hscr.fna

# count seqs in seqs_hscr.fna
count_seqs.py -i hscr/seqs_hscr.fna -o hscr/hscr_count.txt
```

#### Summary of sequence statistics¶

- *hscr\_count.txt* = **982,057** averaging **378bp** in length

### Open-reference OTU picking workflow¶

OTUs are selected using **uclust** with mostly default parameters. The parameters file specifies **pick\_otus:enable\_rev\_strand\_match True**. Any sequences that fail to hit are re-referenced in the reverse orientation to see if a match is obtained. This step will increase in computational time.

**NOTE:** absolute paths must be specified, hence $PWD

In [ ]:

```
# HSCR dataset
pick_open_reference_otus.py -p $PWD/params/OTUpick_params.txt -i $PWD/hscr/seqs_hscr.fna -o $PWD/hscr/open_ref/ 

# C57BL/6J dataset
pick_open_reference_otus.py -p $PWD/params/OTUpick_params.txt -i $PWD/c57/C57BL6J_seqs.fna -o $PWD/c57/open_ref/
```

### Rename OTU tables and phylogenetic trees¶

The pick\_open\_reference\_otus.py produces multiple outputs. We are interested in *otu\_table\_mc2\_w\_tax\_no\_pynast\_failures.biom*. This OTU table contains OTUs with taxonomy and has filtered out any OTUs that did not align to 16S reference sequences with PyNAST. We will copy and rename these tables along with their resepctive phylogenetic trees. The cp command is chosen over the mv command in order to leave the original files untouched.

In [ ]:

```
# HSCR dataset

# copy and rename table and tree
cp hscr/open_ref/otu_table_mc2_w_tax_no_pynast_failures.biom hscr/open_ref/HSCR_OTU_table.biom
cp hscr/open_ref/rep_set.tre hscr/open_ref/HSCR_rep_set.tre

# C57BL/6J dataset

# copy and rename table and tree
cp c57/open_ref/otu_table_mc2_w_tax_no_pynast_failures.biom c57/open_ref/C57_OTU_table.biom
cp c57/open_ref/rep_set.tre c57/open_ref/C57_rep_set.tre
```

### Remove chloroplast sequences from OTU tables¶

We will filter our OTU tables to remove Order Streptophyta, which represents 16S chloroplast rRNA sequences.

In [ ]:

```
# HSCR dataset
filter_taxa_from_otu_table.py -i hscr/open_ref/HSCR_OTU_table.biom -o hscr/open_ref/HSCR_OTU_table.biom -n o__Streptophyta

# C57BL/6J dataset
filter_taxa_from_otu_table.py -i c57/open_ref/C57_OTU_table.biom -o c57/open_ref/C57_OTU_table.biom -n o__Streptophyta
```

### Summarize each master OTU table¶

**NOTE:** The "Num samples" will (and must) always be the same, while the other numbers reported below may vary slightly given updated databases, and OTU clustering methods in general. The numbers in the published OTU tables are listed below.

In [ ]:

```
# HSCR dataset
biom summarize-table -i hscr/open_ref/HSCR_OTU_table.biom -o hscr/open_ref/smry_HSCR_OTU_table.txt

# C57BL/6J dataset
biom summarize-table -i c57/open_ref/C57_OTU_table.biom -o c57/open_ref/smry_C57_OTU_table.txt
```

#### Summary of OTU table statistics¶

*smry\_HSCR\_OTU\_table.txt*

- Num samples: **114**
- Num observations: **8750**
- Total count: **889,947**
- Min: **374**
- Max: **44,836**

*smry\_C57\_OTU\_table.txt*

- Num samples: **20**
- Num observations: **3774**
- Total count: **678,466**
- Min: **12,790**
- Max: **57,020**

##### Achievement unlocked. Proceed to: QIIME\_Code\_2.html¶
