# Supplementary material for: Microbiome Composition in Both Wild-Type and Disease Model Mice Is Heavily Influenced by Mouse Facility
Source: Front Microbiol. 2018 Jul 20;9:1598. doi: 10.3389/fmicb.2018.01598 (PMC6062620; doi:10.3389/fmicb.2018.01598)
Supplement: Supplementary file 2 [file Data_Sheet_2.ZIP › DataSheet2/QIIME_Code_2.html]

QIIME\_Code\_2


## Command-line code for OTU table filtering, rarefaction, and all analyses using QIIME 1.¶

This file includes:

- OTU table filtering
- Rarefaction
- Beta Diversity
- Alpha Diversity
- Core Microbiome Analysis
- Taxonomic Classification
- Differential Abundance Testing

**NOTE:** Choose Option 1 or Option 2 below, based upon how you accessed the data.

### Option 1 (recommended): Setup directories and files - from downloading DataSheet2.zip¶

If you downloaded the files in DataSheet2.zip, follow the steps below to setup the directory hierarchy and file locations in order to run everything in "copy-paste mode." From wherever files are downloaded, copy and rename them to the appropriate locations, specified below.

In [ ]:

```
# change working directory
cd Desktop/

# make new directories
mkdir q1_analysis/
mkdir q1_analysis/hscr/
mkdir q1_analysis/hscr/open_ref/
mkdir q1_analysis/c57/
mkdir q1_analysis/c57/open_ref/

# move files (in this case, they were located in "~/Downloads")
# change working directory
cd ../Downloads

# unzip the folder (and deletes the extra created folder by default)
unzip -q DataSheet2.zip;rm -rf __MACOSX

# copy and rename the data files
cp DataSheet2/HSCR_metadata.txt ../Desktop/q1_analysis/HSCR_metadata.txt
cp DataSheet2/HSCR_OTU_table.biom ../Desktop/q1_analysis/hscr/open_ref/HSCR_OTU_table.biom
cp DataSheet2/HSCR_rep_set.tre ../Desktop/q1_analysis/hscr/open_ref/HSCR_rep_set.tre
cp DataSheet2/C57_metadata.txt ../Desktop/q1_analysis/C57_metadata.txt
cp DataSheet2/C57_OTU_table.biom ../Desktop/q1_analysis/c57/open_ref/C57_OTU_table.biom
cp DataSheet2/C57_rep_set.tre ../Desktop/q1_analysis/c57/open_ref/C57_rep_set.tre
cp DataSheet2/HSCR_diffabund_map.txt ../Desktop/q1_analysis/HSCR_diffabund_map.txt
```

#### When finished you should have the following file locations:¶

File locations:

- ~/Desktop/q1\_analysis/HSCR\_metadata.txt
- ~/Desktop/q1\_analysis/hscr/open\_ref/HSCR\_OTU\_table.biom
- ~/Desktop/q1\_analysis/hscr/open\_ref/HSCR\_rep\_set.tre
- ~/Desktop/q1\_analysis/C57\_metadata.txt
- ~/Desktop/q1\_analysis/c57/open\_ref/C57\_OTU\_table.biom
- ~/Desktop/q1\_analysis/c57/open\_ref/C57\_rep\_set.tre
- ~/Desktop/q1\_analysis/HSCR\_diffabund\_map.txt

##### If everything is in order, proceed to "Activate the QIIME 1..." step¶

### Option 2: Setup directories and files - from running S1\_Code.html¶

If you downloaded the sequence files and performed the commands in QIIME\_Code\_1.html, confirm that you have the following file locations:

File locations:

- ~/Desktop/q1\_analysis/HSCR\_metadata.txt
- ~/Desktop/q1\_analysis/hscr/open\_ref/HSCR\_OTU\_table.biom
- ~/Desktop/q1\_analysis/hscr/open\_ref/HSCR\_rep\_set.tre
- ~/Desktop/q1\_analysis/C57\_metadata.txt
- ~/Desktop/q1\_analysis/c57/open\_ref/C57\_OTU\_table.biom
- ~/Desktop/q1\_analysis/c57/open\_ref/C57\_rep\_set.tre
- ~/Desktop/q1\_analysis/HSCR\_diffabund\_map.txt

##### If everything is in order, proceed to "Activate the QIIME 1..." step¶

### Activate the QIIME 1 environment, change working directory, and make new directories¶

In [ ]:

```
# version: 1.9.1
source activate qiime1

# change working directory (navigate to from wherever you are currently located)
# I am coming from Option 1...
cd ../Desktop/q1_analysis/

# make new directories
mkdir hscr/flt_otus/
mkdir hscr/rfd_otus/
mkdir c57/rfd_otus/
mkdir hscr/rfd_otus_flt/
mkdir hscr/core_comp/
mkdir hscr/core/
mkdir hscr/adiv/
mkdir hscr/adiv/inter/
mkdir hscr/adiv/intra/
mkdir hscr/diff_abund/
mkdir hscr/diff_abund/otus/
mkdir hscr/diff_abund/taxa/
mkdir c57/rfd_otus_flt/
mkdir c57/core_comp/
mkdir c57/core/
mkdir c57/adiv/
```

### Filter the unrarefied master-HSCR OTU table¶

The following groupings are needed for multiple downstream analyses:

- *cBL\_HSCR.biom* = All Boston and Laramie Colon Samples
- *fBL\_HSCR.biom* = All Boston and Laramie Fecal Samples
- *cBos\_HSCR.biom* = Only Boston Colon Samples
- *fBos\_HSCR.biom* = Only Boston Fecal Samples
- *cLar\_HSCR.biom* = Only Laramie Colon Samples
- *fLar\_HSCR.biom* = Only Laramie Fecal Samples

In [ ]:

```
# filter OTU tables using metadata from the mapping file specifying the groups listed above
filter_samples_from_otu_table.py -i hscr/open_ref/HSCR_OTU_table.biom -m HSCR_metadata.txt -o hscr/flt_otus/cBL_HSCR.biom -s 'Type:colon'
filter_samples_from_otu_table.py -i hscr/open_ref/HSCR_OTU_table.biom -m HSCR_metadata.txt -o hscr/flt_otus/fBL_HSCR.biom -s 'Type:fecal'
filter_samples_from_otu_table.py -i hscr/open_ref/HSCR_OTU_table.biom -m HSCR_metadata.txt -o hscr/flt_otus/cBos_HSCR.biom -s 'FacilityType:Boston-C'
filter_samples_from_otu_table.py -i hscr/open_ref/HSCR_OTU_table.biom -m HSCR_metadata.txt -o hscr/flt_otus/fBos_HSCR.biom -s 'FacilityType:Boston-F'
filter_samples_from_otu_table.py -i hscr/open_ref/HSCR_OTU_table.biom -m HSCR_metadata.txt -o hscr/flt_otus/cLar_HSCR.biom -s 'FacilityType:Laramie-C'
filter_samples_from_otu_table.py -i hscr/open_ref/HSCR_OTU_table.biom -m HSCR_metadata.txt -o hscr/flt_otus/fLar_HSCR.biom -s 'FacilityType:Laramie-F'
```

### Summarize each HSCR filtered OTU table¶

**NOTE:** The "Num samples" will (and must) always be the same, while the other numbers reported below may vary slightly given updated databases, and OTU clustering methods in general. The numbers in the published OTU tables are listed below.

In [ ]:

```
# summarize each filtered OTU table
biom summarize-table -i hscr/flt_otus/cBL_HSCR.biom -o hscr/flt_otus/smry_cBL_HSCR.txt
biom summarize-table -i hscr/flt_otus/fBL_HSCR.biom -o hscr/flt_otus/smry_fBL_HSCR.txt
biom summarize-table -i hscr/flt_otus/cBos_HSCR.biom -o hscr/flt_otus/smry_cBos_HSCR.txt
biom summarize-table -i hscr/flt_otus/fBos_HSCR.biom -o hscr/flt_otus/smry_fBos_HSCR.txt
biom summarize-table -i hscr/flt_otus/cLar_HSCR.biom -o hscr/flt_otus/smry_cLar_HSCR.txt
biom summarize-table -i hscr/flt_otus/fLar_HSCR.biom -o hscr/flt_otus/smry_fLar_HSCR.txt
```

#### Summary of OTU table statistics¶

*smry\_cBL\_HSCR.txt*

- Num samples: **58**
- Min: **374**

*smry\_fBL\_HSCR.txt*

- Num samples: **56**
- Min: **396**

*smry\_cBos\_HSCR.txt*

- Num samples: **26**
- Min: **695**

*smry\_fBos\_HSCR.txt*

- Num samples: **26**
- Min: **1791**

*smry\_cLar\_HSCR.txt*

- Num samples: **32**
- Min: **374**

*smry\_fLar\_HSCR.txt*

- Num samples: **30**
- Min: **396**

### Rarefy all OTU tables¶

The value of "Min" from each of the summaries above gives us the rarefaction depth for each OTU table. Since the number of samples per group is low we cannot afford to lose any sample. Therefore, we must rarefy to the sample with the lowest depth in each OTU table.

Rarefaction depths for the published OTU tables are as follows:

HSCR dataset:

- *HSCR\_OTU\_table.biom* = **374**
- *cBL\_HSCR.biom* = **374**
- *fBL\_HSCR.biom* = **396**
- *cBos\_HSCR.biom* = **695**
- *fBos\_HSCR.biom* = **1791**
- *cLar\_HSCR.biom* = **374**
- *fLar\_HSCR.biom* = **396**

For the C57BL/6J dataset:

- *C57\_OTU\_table.biom* = **12790**

In [ ]:

```
# HSCR dataset
single_rarefaction.py -i hscr/open_ref/HSCR_OTU_table.biom -o hscr/rfd_otus/HSCR_OTU_table_rare.biom -d 374
single_rarefaction.py -i hscr/flt_otus/cBL_HSCR.biom -o hscr/rfd_otus/cBL_HSCR_rare.biom -d 374
single_rarefaction.py -i hscr/flt_otus/fBL_HSCR.biom -o hscr/rfd_otus/fBL_HSCR_rare.biom -d 396
single_rarefaction.py -i hscr/flt_otus/cBos_HSCR.biom -o hscr/rfd_otus/cBos_HSCR_rare.biom -d 695
single_rarefaction.py -i hscr/flt_otus/fBos_HSCR.biom -o hscr/rfd_otus/fBos_HSCR_rare.biom -d 1791
single_rarefaction.py -i hscr/flt_otus/cLar_HSCR.biom -o hscr/rfd_otus/cLar_HSCR_rare.biom -d 374
single_rarefaction.py -i hscr/flt_otus/fLar_HSCR.biom -o hscr/rfd_otus/fLar_HSCR_rare.biom -d 396

# C57BL/6J dataset
single_rarefaction.py -i c57/open_ref/C57_OTU_table.biom -o c57/rfd_otus/C57_OTU_table_rare.biom -d 12790
```

### Calculate total microbiome beta diversity¶

Using rarefied OTU tables, calculate unweighted and weighted UniFrac (the defaults for this script). This script can be run in batch on a directory, which is rad.

In [ ]:

```
# HSCR dataset
beta_diversity.py -i hscr/rfd_otus/ -t hscr/open_ref/HSCR_rep_set.tre -o hscr/bdiv/

# C57BL/6J dataset
beta_diversity.py -i c57/rfd_otus/ -t c57/open_ref/C57_rep_set.tre -o c57/bdiv/
```

### Compute core microbiomes and calculate core microbiome beta diversity¶

Using rarefied OTU tables, this analysis identifies core OTUs, defined as OTUs present in at least x% of samples, where x is the chosen percentage threshold: 50-75-100. For both datasets, first filter rarefied OTU tables for the appropriate groups. We will compare individual facility (i.e. Boston or Laramie ) core microbiomes to "conserved" core microbiomes calculated for both facilites together (i.e. Boston and Laramie). We will calculate beta diversity - weighted UniFrac only - on the conserved core microbiomes only.

**NOTE** The compute\_core\_microbiome.py script may cause an error ending with: "Attempting to write an empty BIOM table to disk. QIIME doesn't support writing empty BIOM output files." Disregard this error. If no OTUs are found to be present in the core thresholds defined above, no OTU table can be created. This happens almost exclusively at the 100 threshold.

In [ ]:

```
# HSCR dataset
# filter samples
filter_samples_from_otu_table.py -m HSCR_metadata.txt -i hscr/rfd_otus/cBL_HSCR_rare.biom -o hscr/rfd_otus_flt/cBL_P07WT.biom -s 'Group:P07-WT'
filter_samples_from_otu_table.py -m HSCR_metadata.txt -i hscr/rfd_otus/cBL_HSCR_rare.biom -o hscr/rfd_otus_flt/cBos_P07WT.biom -s 'GroupFacility:P07-WT-Bos'
filter_samples_from_otu_table.py -m HSCR_metadata.txt -i hscr/rfd_otus/cBL_HSCR_rare.biom -o hscr/rfd_otus_flt/cLar_P07WT.biom -s 'GroupFacility:P07-WT-Lar'
filter_samples_from_otu_table.py -m HSCR_metadata.txt -i hscr/rfd_otus/cBL_HSCR_rare.biom -o hscr/rfd_otus_flt/cBL_P07KO.biom -s 'Group:P07-KO'
filter_samples_from_otu_table.py -m HSCR_metadata.txt -i hscr/rfd_otus/cBL_HSCR_rare.biom -o hscr/rfd_otus_flt/cBos_P07KO.biom -s 'GroupFacility:P07-KO-Bos'
filter_samples_from_otu_table.py -m HSCR_metadata.txt -i hscr/rfd_otus/cBL_HSCR_rare.biom -o hscr/rfd_otus_flt/cLar_P07KO.biom -s 'GroupFacility:P07-KO-Lar'

filter_samples_from_otu_table.py -m HSCR_metadata.txt -i hscr/rfd_otus/cBL_HSCR_rare.biom -o hscr/rfd_otus_flt/cBL_P20WT.biom -s 'Group:P20-WT'
filter_samples_from_otu_table.py -m HSCR_metadata.txt -i hscr/rfd_otus/cBL_HSCR_rare.biom -o hscr/rfd_otus_flt/cBos_P20WT.biom -s 'GroupFacility:P20-WT-Bos'
filter_samples_from_otu_table.py -m HSCR_metadata.txt -i hscr/rfd_otus/cBL_HSCR_rare.biom -o hscr/rfd_otus_flt/cLar_P20WT.biom -s 'GroupFacility:P20-WT-Lar'
filter_samples_from_otu_table.py -m HSCR_metadata.txt -i hscr/rfd_otus/cBL_HSCR_rare.biom -o hscr/rfd_otus_flt/cBL_P20KO.biom -s 'Group:P20-KO'
filter_samples_from_otu_table.py -m HSCR_metadata.txt -i hscr/rfd_otus/cBL_HSCR_rare.biom -o hscr/rfd_otus_flt/cBos_P20KO.biom -s 'GroupFacility:P20-KO-Bos'
filter_samples_from_otu_table.py -m HSCR_metadata.txt -i hscr/rfd_otus/cBL_HSCR_rare.biom -o hscr/rfd_otus_flt/cLar_P20KO.biom -s 'GroupFacility:P20-KO-Lar'
filter_samples_from_otu_table.py -m HSCR_metadata.txt -i hscr/rfd_otus/cBL_HSCR_rare.biom -o hscr/rfd_otus_flt/cLar_P20KO.biom -s 'GroupFacility:P20-KO-Lar'

filter_samples_from_otu_table.py -m HSCR_metadata.txt -i hscr/rfd_otus/cBL_HSCR_rare.biom -o hscr/rfd_otus_flt/cBL_P24WT.biom -s 'Group:P24-WT'
filter_samples_from_otu_table.py -m HSCR_metadata.txt -i hscr/rfd_otus/cBL_HSCR_rare.biom -o hscr/rfd_otus_flt/cBos_P24WT.biom -s 'GroupFacility:P24-WT-Bos'
filter_samples_from_otu_table.py -m HSCR_metadata.txt -i hscr/rfd_otus/cBL_HSCR_rare.biom -o hscr/rfd_otus_flt/cLar_P24WT.biom -s 'GroupFacility:P24-WT-Lar'
filter_samples_from_otu_table.py -m HSCR_metadata.txt -i hscr/rfd_otus/cBL_HSCR_rare.biom -o hscr/rfd_otus_flt/cBL_P24KO.biom -s 'Group:P24-KO'
filter_samples_from_otu_table.py -m HSCR_metadata.txt -i hscr/rfd_otus/cBL_HSCR_rare.biom -o hscr/rfd_otus_flt/cBos_P24KO.biom -s 'GroupFacility:P24-KO-Bos'
filter_samples_from_otu_table.py -m HSCR_metadata.txt -i hscr/rfd_otus/cBL_HSCR_rare.biom -o hscr/rfd_otus_flt/cLar_P24KO.biom -s 'GroupFacility:P24-KO-Lar'

filter_samples_from_otu_table.py -m HSCR_metadata.txt -i hscr/rfd_otus/fBL_HSCR_rare.biom -o hscr/rfd_otus_flt/fBL_P07WT.biom -s 'Group:P07-WT'
filter_samples_from_otu_table.py -m HSCR_metadata.txt -i hscr/rfd_otus/fBL_HSCR_rare.biom -o hscr/rfd_otus_flt/fBos_P07WT.biom -s 'GroupFacility:P07-WT-Bos'
filter_samples_from_otu_table.py -m HSCR_metadata.txt -i hscr/rfd_otus/fBL_HSCR_rare.biom -o hscr/rfd_otus_flt/fLar_P07WT.biom -s 'GroupFacility:P07-WT-Lar'
filter_samples_from_otu_table.py -m HSCR_metadata.txt -i hscr/rfd_otus/fBL_HSCR_rare.biom -o hscr/rfd_otus_flt/fBL_P07KO.biom -s 'Group:P07-KO'
filter_samples_from_otu_table.py -m HSCR_metadata.txt -i hscr/rfd_otus/fBL_HSCR_rare.biom -o hscr/rfd_otus_flt/fBos_P07KO.biom -s 'GroupFacility:P07-KO-Bos'
filter_samples_from_otu_table.py -m HSCR_metadata.txt -i hscr/rfd_otus/fBL_HSCR_rare.biom -o hscr/rfd_otus_flt/fLar_P07KO.biom -s 'GroupFacility:P07-KO-Lar'

filter_samples_from_otu_table.py -m HSCR_metadata.txt -i hscr/rfd_otus/fBL_HSCR_rare.biom -o hscr/rfd_otus_flt/fBL_P20WT.biom -s 'Group:P20-WT'
filter_samples_from_otu_table.py -m HSCR_metadata.txt -i hscr/rfd_otus/fBL_HSCR_rare.biom -o hscr/rfd_otus_flt/fBos_P20WT.biom -s 'GroupFacility:P20-WT-Bos'
filter_samples_from_otu_table.py -m HSCR_metadata.txt -i hscr/rfd_otus/fBL_HSCR_rare.biom -o hscr/rfd_otus_flt/fLar_P20WT.biom -s 'GroupFacility:P20-WT-Lar'
filter_samples_from_otu_table.py -m HSCR_metadata.txt -i hscr/rfd_otus/fBL_HSCR_rare.biom -o hscr/rfd_otus_flt/fBL_P20KO.biom -s 'Group:P20-KO'
filter_samples_from_otu_table.py -m HSCR_metadata.txt -i hscr/rfd_otus/fBL_HSCR_rare.biom -o hscr/rfd_otus_flt/fBos_P20KO.biom -s 'GroupFacility:P20-KO-Bos'
filter_samples_from_otu_table.py -m HSCR_metadata.txt -i hscr/rfd_otus/fBL_HSCR_rare.biom -o hscr/rfd_otus_flt/fLar_P20KO.biom -s 'GroupFacility:P20-KO-Lar'

filter_samples_from_otu_table.py -m HSCR_metadata.txt -i hscr/rfd_otus/fBL_HSCR_rare.biom -o hscr/rfd_otus_flt/fBL_P24WT.biom -s 'Group:P24-WT'
filter_samples_from_otu_table.py -m HSCR_metadata.txt -i hscr/rfd_otus/fBL_HSCR_rare.biom -o hscr/rfd_otus_flt/fBos_P24WT.biom -s 'GroupFacility:P24-WT-Bos'
filter_samples_from_otu_table.py -m HSCR_metadata.txt -i hscr/rfd_otus/fBL_HSCR_rare.biom -o hscr/rfd_otus_flt/fLar_P24WT.biom -s 'GroupFacility:P24-WT-Lar'
filter_samples_from_otu_table.py -m HSCR_metadata.txt -i hscr/rfd_otus/fBL_HSCR_rare.biom -o hscr/rfd_otus_flt/fBL_P24KO.biom -s 'Group:P24-KO'
filter_samples_from_otu_table.py -m HSCR_metadata.txt -i hscr/rfd_otus/fBL_HSCR_rare.biom -o hscr/rfd_otus_flt/fBos_P24KO.biom -s 'GroupFacility:P24-KO-Bos'
filter_samples_from_otu_table.py -m HSCR_metadata.txt -i hscr/rfd_otus/fBL_HSCR_rare.biom -o hscr/rfd_otus_flt/fLar_P24KO.biom -s 'GroupFacility:P24-KO-Lar'

# compute core microbiomes
compute_core_microbiome.py --max_fraction_for_core 1.0 --min_fraction_for_core 0.5 --num_fraction_for_core_steps 3 -i hscr/rfd_otus_flt/cBL_P07WT.biom -o hscr/core_comp/cBL_P07WT/
compute_core_microbiome.py --max_fraction_for_core 1.0 --min_fraction_for_core 0.5 --num_fraction_for_core_steps 3 -i hscr/rfd_otus_flt/cLar_P07WT.biom -o hscr/core_comp/cLar_P07WT/
compute_core_microbiome.py --max_fraction_for_core 1.0 --min_fraction_for_core 0.5 --num_fraction_for_core_steps 3 -i hscr/rfd_otus_flt/cBos_P07WT.biom -o hscr/core_comp/cBos_P07WT/
compute_core_microbiome.py --max_fraction_for_core 1.0 --min_fraction_for_core 0.5 --num_fraction_for_core_steps 3 -i hscr/rfd_otus_flt/cBL_P07KO.biom -o hscr/core_comp/cBL_P07KO/
compute_core_microbiome.py --max_fraction_for_core 1.0 --min_fraction_for_core 0.5 --num_fraction_for_core_steps 3 -i hscr/rfd_otus_flt/cBos_P07KO.biom -o hscr/core_comp/cBos_P07KO/
compute_core_microbiome.py --max_fraction_for_core 1.0 --min_fraction_for_core 0.5 --num_fraction_for_core_steps 3 -i hscr/rfd_otus_flt/cLar_P07KO.biom -o hscr/core_comp/cLar_P07KO/

compute_core_microbiome.py --max_fraction_for_core 1.0 --min_fraction_for_core 0.5 --num_fraction_for_core_steps 3 -i hscr/rfd_otus_flt/cBL_P20WT.biom -o hscr/core_comp/cBL_P20WT/
compute_core_microbiome.py --max_fraction_for_core 1.0 --min_fraction_for_core 0.5 --num_fraction_for_core_steps 3 -i hscr/rfd_otus_flt/cBos_P20WT.biom -o hscr/core_comp/cBos_P20WT/
compute_core_microbiome.py --max_fraction_for_core 1.0 --min_fraction_for_core 0.5 --num_fraction_for_core_steps 3 -i hscr/rfd_otus_flt/cLar_P20WT.biom -o hscr/core_comp/cLar_P20WT/
compute_core_microbiome.py --max_fraction_for_core 1.0 --min_fraction_for_core 0.5 --num_fraction_for_core_steps 3 -i hscr/rfd_otus_flt/cBL_P20KO.biom -o hscr/core_comp/cBL_P20KO/
compute_core_microbiome.py --max_fraction_for_core 1.0 --min_fraction_for_core 0.5 --num_fraction_for_core_steps 3 -i hscr/rfd_otus_flt/cBos_P20KO.biom -o hscr/core_comp/cBos_P20KO/
compute_core_microbiome.py --max_fraction_for_core 1.0 --min_fraction_for_core 0.5 --num_fraction_for_core_steps 3 -i hscr/rfd_otus_flt/cLar_P20KO.biom -o hscr/core_comp/cLar_P20KO/

compute_core_microbiome.py --max_fraction_for_core 1.0 --min_fraction_for_core 0.5 --num_fraction_for_core_steps 3 -i hscr/rfd_otus_flt/cBL_P24WT.biom -o hscr/core_comp/cBL_P24WT/
compute_core_microbiome.py --max_fraction_for_core 1.0 --min_fraction_for_core 0.5 --num_fraction_for_core_steps 3 -i hscr/rfd_otus_flt/cBos_P24WT.biom -o hscr/core_comp/cBos_P24WT/
compute_core_microbiome.py --max_fraction_for_core 1.0 --min_fraction_for_core 0.5 --num_fraction_for_core_steps 3 -i hscr/rfd_otus_flt/cLar_P24WT.biom -o hscr/core_comp/cLar_P24WT/
compute_core_microbiome.py --max_fraction_for_core 1.0 --min_fraction_for_core 0.5 --num_fraction_for_core_steps 3 -i hscr/rfd_otus_flt/cBL_P24KO.biom -o hscr/core_comp/cBL_P24KO/
compute_core_microbiome.py --max_fraction_for_core 1.0 --min_fraction_for_core 0.5 --num_fraction_for_core_steps 3 -i hscr/rfd_otus_flt/cBos_P24KO.biom -o hscr/core_comp/cBos_P24KO/
compute_core_microbiome.py --max_fraction_for_core 1.0 --min_fraction_for_core 0.5 --num_fraction_for_core_steps 3 -i hscr/rfd_otus_flt/cLar_P24KO.biom -o hscr/core_comp/cLar_P24KO/

compute_core_microbiome.py --max_fraction_for_core 1.0 --min_fraction_for_core 0.5 --num_fraction_for_core_steps 3 -i hscr/rfd_otus_flt/fBL_P07WT.biom -o hscr/core_comp/fBL_P07WT/
compute_core_microbiome.py --max_fraction_for_core 1.0 --min_fraction_for_core 0.5 --num_fraction_for_core_steps 3 -i hscr/rfd_otus_flt/fBos_P07WT.biom -o hscr/core_comp/fBos_P07WT/
compute_core_microbiome.py --max_fraction_for_core 1.0 --min_fraction_for_core 0.5 --num_fraction_for_core_steps 3 -i hscr/rfd_otus_flt/fLar_P07WT.biom -o hscr/core_comp/fLar_P07WT/
compute_core_microbiome.py --max_fraction_for_core 1.0 --min_fraction_for_core 0.5 --num_fraction_for_core_steps 3 -i hscr/rfd_otus_flt/fBL_P07KO.biom -o hscr/core_comp/fBL_P07KO/
compute_core_microbiome.py --max_fraction_for_core 1.0 --min_fraction_for_core 0.5 --num_fraction_for_core_steps 3 -i hscr/rfd_otus_flt/fBos_P07KO.biom -o hscr/core_comp/fBos_P07KO/
compute_core_microbiome.py --max_fraction_for_core 1.0 --min_fraction_for_core 0.5 --num_fraction_for_core_steps 3 -i hscr/rfd_otus_flt/fLar_P07KO.biom -o hscr/core_comp/fLar_P07KO/

compute_core_microbiome.py --max_fraction_for_core 1.0 --min_fraction_for_core 0.5 --num_fraction_for_core_steps 3 -i hscr/rfd_otus_flt/fBL_P20WT.biom -o hscr/core_comp/fBL_P20WT/
compute_core_microbiome.py --max_fraction_for_core 1.0 --min_fraction_for_core 0.5 --num_fraction_for_core_steps 3 -i hscr/rfd_otus_flt/fBos_P20WT.biom -o hscr/core_comp/fBos_P20WT/
compute_core_microbiome.py --max_fraction_for_core 1.0 --min_fraction_for_core 0.5 --num_fraction_for_core_steps 3 -i hscr/rfd_otus_flt/fLar_P20WT.biom -o hscr/core_comp/fLar_P20WT/
compute_core_microbiome.py --max_fraction_for_core 1.0 --min_fraction_for_core 0.5 --num_fraction_for_core_steps 3 -i hscr/rfd_otus_flt/fBL_P20KO.biom -o hscr/core_comp/fBL_P20KO/
compute_core_microbiome.py --max_fraction_for_core 1.0 --min_fraction_for_core 0.5 --num_fraction_for_core_steps 3 -i hscr/rfd_otus_flt/fBos_P20KO.biom -o hscr/core_comp/fBos_P20KO/
compute_core_microbiome.py --max_fraction_for_core 1.0 --min_fraction_for_core 0.5 --num_fraction_for_core_steps 3 -i hscr/rfd_otus_flt/fLar_P20KO.biom -o hscr/core_comp/fLar_P20KO/

compute_core_microbiome.py --max_fraction_for_core 1.0 --min_fraction_for_core 0.5 --num_fraction_for_core_steps 3 -i hscr/rfd_otus_flt/fBL_P24WT.biom -o hscr/core_comp/fBL_P24WT/
compute_core_microbiome.py --max_fraction_for_core 1.0 --min_fraction_for_core 0.5 --num_fraction_for_core_steps 3 -i hscr/rfd_otus_flt/fBos_P24WT.biom -o hscr/core_comp/fBos_P24WT/
compute_core_microbiome.py --max_fraction_for_core 1.0 --min_fraction_for_core 0.5 --num_fraction_for_core_steps 3 -i hscr/rfd_otus_flt/fLar_P24WT.biom -o hscr/core_comp/fLar_P24WT/
compute_core_microbiome.py --max_fraction_for_core 1.0 --min_fraction_for_core 0.5 --num_fraction_for_core_steps 3 -i hscr/rfd_otus_flt/fBL_P24KO.biom -o hscr/core_comp/fBL_P24KO/
compute_core_microbiome.py --max_fraction_for_core 1.0 --min_fraction_for_core 0.5 --num_fraction_for_core_steps 3 -i hscr/rfd_otus_flt/fBos_P24KO.biom -o hscr/core_comp/fBos_P24KO/
compute_core_microbiome.py --max_fraction_for_core 1.0 --min_fraction_for_core 0.5 --num_fraction_for_core_steps 3 -i hscr/rfd_otus_flt/fLar_P24KO.biom -o hscr/core_comp/fLar_P24KO/

# copy, move, rename the appropriate files
cp hscr/core_comp/cBL_P07WT/core_table_50.biom hscr/core/cBL_P07WTcore50.biom
cp hscr/core_comp/fBL_P07WT/core_table_50.biom hscr/core/fBL_P07WTcore50.biom
cp hscr/core_comp/cBL_P07KO/core_table_50.biom hscr/core/cBL_P07KOcore50.biom
cp hscr/core_comp/fBL_P07KO/core_table_50.biom hscr/core/fBL_P07KOcore50.biom
cp hscr/core_comp/cBL_P20WT/core_table_50.biom hscr/core/cBL_P20WTcore50.biom
cp hscr/core_comp/fBL_P20WT/core_table_50.biom hscr/core/fBL_P20WTcore50.biom
cp hscr/core_comp/cBL_P20KO/core_table_50.biom hscr/core/cBL_P20KOcore50.biom
cp hscr/core_comp/fBL_P20KO/core_table_50.biom hscr/core/fBL_P20KOcore50.biom
cp hscr/core_comp/cBL_P24WT/core_table_50.biom hscr/core/cBL_P24WTcore50.biom
cp hscr/core_comp/fBL_P24WT/core_table_50.biom hscr/core/fBL_P24WTcore50.biom
cp hscr/core_comp/cBL_P24KO/core_table_50.biom hscr/core/cBL_P24KOcore50.biom
cp hscr/core_comp/fBL_P24KO/core_table_50.biom hscr/core/fBL_P24KOcore50.biom

# calculate beta diversity (weighted UniFrac only) on core/ directory
beta_diversity.py -i hscr/core/ -t hscr/open_ref/HSCR_rep_set.tre -m weighted_unifrac -o hscr/core_bdiv/

# C57BL/6J dataset
# filter samples
filter_samples_from_otu_table.py -m C57_metadata.txt -i c57/rfd_otus/C57_OTU_table_rare.biom -o c57/rfd_otus_flt/bos_c57_rare.biom -s 'Facility:Boston'
filter_samples_from_otu_table.py -m C57_metadata.txt -i c57/rfd_otus/C57_OTU_table_rare.biom -o c57/rfd_otus_flt/lar_c57_rare.biom -s 'Facility:Laramie'

# compute core microbiomes
compute_core_microbiome.py --max_fraction_for_core 1.0 --min_fraction_for_core 0.5 --num_fraction_for_core_steps 3 -i c57/rfd_otus/C57_OTU_table_rare.biom -o c57/core_comp/BL_c57/
compute_core_microbiome.py --max_fraction_for_core 1.0 --min_fraction_for_core 0.5 --num_fraction_for_core_steps 3 -i c57/rfd_otus_flt/bos_c57_rare.biom -o c57/core_comp/bos_c57/
compute_core_microbiome.py --max_fraction_for_core 1.0 --min_fraction_for_core 0.5 --num_fraction_for_core_steps 3 -i c57/rfd_otus_flt/lar_c57_rare.biom -o c57/core_comp/lar_c57/

# copy, move, rename the appropriate file generated above
cp c57/core_comp/BL_c57/core_table_50.biom c57/core/c57core50.biom

# calculate beta diversity (weighted unifrac only) on core/ directory
beta_diversity.py -i c57/core/ -t c57/open_ref/C57_rep_set.tre -m weighted_unifrac -o c57/core_bdiv/
```

### Summarize taxonomic information¶

We will 'extract' the taxonomic information from the OTU tables. For this, we are using relative abundances and therefore it is recommended to use raw (unrarefied OTU tables).

**NOTE**: genus level info is not needed for the C57BL/6J dataset.

In [ ]:

```
# Phylum
# HSCR dataset
summarize_taxa.py -m HSCR_metadata.txt -i hscr/open_ref/HSCR_OTU_table.biom -o hscr/taxa/ -L 2

# copy and rename the file
cp hscr/taxa/HSCR_metadata_L2.txt hscr/taxa/HSCR_phylum.txt

# Genus
# HSCR dataset
summarize_taxa.py -m HSCR_metadata.txt -i hscr/open_ref/HSCR_OTU_table.biom -o hscr/taxa/ -L 6

# copy and rename the file
cp hscr/taxa/HSCR_metadata_L6.txt hscr/taxa/HSCR_genus.txt

# C57BL/6J dataset
summarize_taxa.py -m C57_metadata.txt -i c57/open_ref/C57_OTU_table.biom -o c57/taxa/ -L 2

# copy and rename the file
cp c57/taxa/C57_metadata.txt_L2.txt c57/taxa/c57_phylum.txt
```

### Calculate alpha diversity: Chao1 and observed OTUs¶

Using rarefied OTU tables, we will calculate Chao1 and observed OTUs alpha diversity metrics.

In [ ]:

```
# HSCR dataset
# chao1 and observed_otus
alpha_diversity.py -m chao1,observed_otus -i hscr/rfd_otus/cBL_HSCR_rare.biom -o hscr/adiv/inter/cBL_HSCR_adiv.txt
alpha_diversity.py -m chao1,observed_otus -i hscr/rfd_otus/fBL_HSCR_rare.biom -o hscr/adiv/inter/fBL_HSCR_adiv.txt
alpha_diversity.py -m chao1,observed_otus -i hscr/rfd_otus/cBos_HSCR_rare.biom -o hscr/adiv/intra/cBos_HSCR_adiv.txt
alpha_diversity.py -m chao1,observed_otus -i hscr/rfd_otus/fBos_HSCR_rare.biom -o hscr/adiv/intra/fBos_HSCR_adiv.txt
alpha_diversity.py -m chao1,observed_otus -i hscr/rfd_otus/cLar_HSCR_rare.biom -o hscr/adiv/intra/cLar_HSCR_adiv.txt
alpha_diversity.py -m chao1,observed_otus -i hscr/rfd_otus/fLar_HSCR_rare.biom -o hscr/adiv/intra/fLar_HSCR_adiv.txt

# C57BL/6J dataset
# chao1 and observed_otus
alpha_diversity.py -m chao1,observed_otus -i c57/rfd_otus/C57_OTU_table_rare.biom -o c57/adiv/c57_adiv.txt
```

### Calculate Differential Abundance of OTUs and Taxa¶

Using rarefied OTU tables and relative abundance biom tables from the HSCR dataset, we will perform differential abundance testing. We are looking for OTUs or taxa that are over- or under-represented in one genotype or the other (aka associated with only KO- or only WT-mice). For OTUs, kruskal wallis, nonparametric T test, and g-test will be used. For taxa, the kruskal wallis and nonparametric T test will be used. The g-test will not accept relative abundance values.

A special map, diffabund\_map.txt, was created for this analysis. This map has columns specifying certain groups, leaving the rest empty. For instance, column 'cBos\_Age07' contains rows with values **ONLY** for P07-KO and P07-WT Boston colon samples. The group\_significance.py script recognizes rows within a column that contain a value and disregards any empty rows. Therefore, we can use the special map to specify the group of interest and use master\_HSCR\_rare.biom for all OTU-based tests, rather than meticulously filtering each OTU table for the group of interest.

**NOTE:** When testing taxa the following warning appears: "No metadata in biom table. Won't alter calculations." This warning can be disregarded. The taxa biom table contains SampleIDs, which will cross-reference with the special map to achieve the proper comparisons.

In [ ]:

```
# OTUs
# HSCR-Boston
# colon
group_significance.py -s kruskal_wallis -c cBos_Age07 -o hscr/diff_abund/otus/cBos_P07_KW.txt -m HSCR_diffabund_map.txt -i hscr/rfd_otus/HSCR_OTU_table_rare.biom 
group_significance.py -s kruskal_wallis -c cBos_Age20 -o hscr/diff_abund/otus/cBos_P20_KW.txt -m HSCR_diffabund_map.txt -i hscr/rfd_otus/HSCR_OTU_table_rare.biom 
group_significance.py -s kruskal_wallis -c cBos_Age24 -o hscr/diff_abund/otus/cBos_P24_KW.txt -m HSCR_diffabund_map.txt -i hscr/rfd_otus/HSCR_OTU_table_rare.biom 

group_significance.py -s nonparametric_t_test -c cBos_Age07 -o hscr/diff_abund/otus/cBos_P07_NPT.txt -m HSCR_diffabund_map.txt -i hscr/rfd_otus/HSCR_OTU_table_rare.biom --permutations 1000 
group_significance.py -s nonparametric_t_test -c cBos_Age20 -o hscr/diff_abund/otus/cBos_P20_NPT.txt -m HSCR_diffabund_map.txt -i hscr/rfd_otus/HSCR_OTU_table_rare.biom --permutations 1000
group_significance.py -s nonparametric_t_test -c cBos_Age24 -o hscr/diff_abund/otus/cBos_P24_NPT.txt -m HSCR_diffabund_map.txt -i hscr/rfd_otus/HSCR_OTU_table_rare.biom --permutations 1000

group_significance.py -s g_test -c cBos_Age07 -o hscr/diff_abund/otus/cBos_P07_GT.txt -m HSCR_diffabund_map.txt -i hscr/rfd_otus/HSCR_OTU_table_rare.biom 
group_significance.py -s g_test -c cBos_Age20 -o hscr/diff_abund/otus/cBos_P20_GT.txt -m HSCR_diffabund_map.txt -i hscr/rfd_otus/HSCR_OTU_table_rare.biom 
group_significance.py -s g_test -c cBos_Age24 -o hscr/diff_abund/otus/cBos_P24_GT.txt -m HSCR_diffabund_map.txt -i hscr/rfd_otus/HSCR_OTU_table_rare.biom 

# fecal
group_significance.py -s kruskal_wallis -c fBos_Age07 -o hscr/diff_abund/otus/fBos_P07_KW.txt -m HSCR_diffabund_map.txt -i hscr/rfd_otus/HSCR_OTU_table_rare.biom 
group_significance.py -s kruskal_wallis -c fBos_Age20 -o hscr/diff_abund/otus/fBos_P20_KW.txt -m HSCR_diffabund_map.txt -i hscr/rfd_otus/HSCR_OTU_table_rare.biom 
group_significance.py -s kruskal_wallis -c fBos_Age24 -o hscr/diff_abund/otus/fBos_P24_KW.txt -m HSCR_diffabund_map.txt -i hscr/rfd_otus/HSCR_OTU_table_rare.biom

group_significance.py -s nonparametric_t_test -c fBos_Age07 -o hscr/diff_abund/otus/fBos_P07_NPT.txt -m HSCR_diffabund_map.txt -i hscr/rfd_otus/HSCR_OTU_table_rare.biom --permutations 1000 
group_significance.py -s nonparametric_t_test -c fBos_Age20 -o hscr/diff_abund/otus/fBos_P20_NPT.txt -m HSCR_diffabund_map.txt -i hscr/rfd_otus/HSCR_OTU_table_rare.biom --permutations 1000
group_significance.py -s nonparametric_t_test -c fBos_Age24 -o hscr/diff_abund/otus/fBos_P24_NPT.txt -m HSCR_diffabund_map.txt -i hscr/rfd_otus/HSCR_OTU_table_rare.biom --permutations 1000

group_significance.py -s g_test -c fBos_Age07 -o hscr/diff_abund/otus/fBos_P07_GT.txt -m HSCR_diffabund_map.txt -i hscr/rfd_otus/HSCR_OTU_table_rare.biom 
group_significance.py -s g_test -c fBos_Age20 -o hscr/diff_abund/otus/fBos_P20_GT.txt -m HSCR_diffabund_map.txt -i hscr/rfd_otus/HSCR_OTU_table_rare.biom 
group_significance.py -s g_test -c fBos_Age24 -o hscr/diff_abund/otus/fBos_P24_GT.txt -m HSCR_diffabund_map.txt -i hscr/rfd_otus/HSCR_OTU_table_rare.biom 

# HSCR-Laramie
# colon
group_significance.py -s kruskal_wallis -c cLar_Age07 -o hscr/diff_abund/otus/cLar_P07_KW.txt -m HSCR_diffabund_map.txt -i hscr/rfd_otus/HSCR_OTU_table_rare.biom 
group_significance.py -s kruskal_wallis -c cLar_Age20 -o hscr/diff_abund/otus/cLar_P20_KW.txt -m HSCR_diffabund_map.txt -i hscr/rfd_otus/HSCR_OTU_table_rare.biom 
group_significance.py -s kruskal_wallis -c cLar_Age24 -o hscr/diff_abund/otus/cLar_P24_KW.txt -m HSCR_diffabund_map.txt -i hscr/rfd_otus/HSCR_OTU_table_rare.biom 

group_significance.py -s nonparametric_t_test -c cLar_Age07 -o hscr/diff_abund/otus/cLar_P07_NPT.txt -m HSCR_diffabund_map.txt -i hscr/rfd_otus/HSCR_OTU_table_rare.biom --permutations 1000 
group_significance.py -s nonparametric_t_test -c cLar_Age20 -o hscr/diff_abund/otus/cLar_P20_NPT.txt -m HSCR_diffabund_map.txt -i hscr/rfd_otus/HSCR_OTU_table_rare.biom --permutations 1000
group_significance.py -s nonparametric_t_test -c cLar_Age24 -o hscr/diff_abund/otus/cLar_P24_NPT.txt -m HSCR_diffabund_map.txt -i hscr/rfd_otus/HSCR_OTU_table_rare.biom --permutations 1000

group_significance.py -s g_test -c cLar_Age07 -o hscr/diff_abund/otus/cLar_P07_GT.txt -m HSCR_diffabund_map.txt -i hscr/rfd_otus/HSCR_OTU_table_rare.biom 
group_significance.py -s g_test -c cLar_Age20 -o hscr/diff_abund/otus/cLar_P20_GT.txt -m HSCR_diffabund_map.txt -i hscr/rfd_otus/HSCR_OTU_table_rare.biom 
group_significance.py -s g_test -c cLar_Age24 -o hscr/diff_abund/otus/cLar_P24_GT.txt -m HSCR_diffabund_map.txt -i hscr/rfd_otus/HSCR_OTU_table_rare.biom 

# fecal
group_significance.py -s kruskal_wallis -c fLar_Age07 -o hscr/diff_abund/otus/fLar_P07_KW.txt -m HSCR_diffabund_map.txt -i hscr/rfd_otus/HSCR_OTU_table_rare.biom 
group_significance.py -s kruskal_wallis -c fLar_Age20 -o hscr/diff_abund/otus/fLar_P20_KW.txt -m HSCR_diffabund_map.txt -i hscr/rfd_otus/HSCR_OTU_table_rare.biom 
group_significance.py -s kruskal_wallis -c fLar_Age24 -o hscr/diff_abund/otus/fLar_P24_KW.txt -m HSCR_diffabund_map.txt -i hscr/rfd_otus/HSCR_OTU_table_rare.biom

group_significance.py -s nonparametric_t_test -c fLar_Age07 -o hscr/diff_abund/otus/fLar_P07_NPT.txt -m HSCR_diffabund_map.txt -i hscr/rfd_otus/HSCR_OTU_table_rare.biom --permutations 1000 
group_significance.py -s nonparametric_t_test -c fLar_Age20 -o hscr/diff_abund/otus/fLar_P20_NPT.txt -m HSCR_diffabund_map.txt -i hscr/rfd_otus/HSCR_OTU_table_rare.biom --permutations 1000
group_significance.py -s nonparametric_t_test -c fLar_Age24 -o hscr/diff_abund/otus/fLar_P24_NPT.txt -m HSCR_diffabund_map.txt -i hscr/rfd_otus/HSCR_OTU_table_rare.biom --permutations 1000

group_significance.py -s g_test -c fLar_Age07 -o hscr/diff_abund/otus/fLar_P07_GT.txt -m HSCR_diffabund_map.txt -i hscr/rfd_otus/HSCR_OTU_table_rare.biom 
group_significance.py -s g_test -c fLar_Age20 -o hscr/diff_abund/otus/fLar_P20_GT.txt -m HSCR_diffabund_map.txt -i hscr/rfd_otus/HSCR_OTU_table_rare.biom 
group_significance.py -s g_test -c fLar_Age24 -o hscr/diff_abund/otus/fLar_P24_GT.txt -m HSCR_diffabund_map.txt -i hscr/rfd_otus/HSCR_OTU_table_rare.biom 

# For taxa, we need to create biom format taxa table
summarize_taxa.py --suppress_classic_table_output -i hscr/open_ref/HSCR_OTU_table.biom -L 2 -o hscr/taxa/
summarize_taxa.py --suppress_classic_table_output -i hscr/open_ref/HSCR_OTU_table.biom -L 6 -o hscr/taxa/

# Taxa
# HSCR-Boston
# phylum (L2)
# colon
group_significance.py -s kruskal_wallis -c cBos_Age07 -o hscr/diff_abund/taxa/cBos_P07_KW_L2.txt -m HSCR_diffabund_map.txt -i hscr/taxa/HSCR_OTU_table_L2.biom
group_significance.py -s kruskal_wallis -c cBos_Age20 -o hscr/diff_abund/taxa/cBos_P20_KW_L2.txt -m HSCR_diffabund_map.txt -i hscr/taxa/HSCR_OTU_table_L2.biom
group_significance.py -s kruskal_wallis -c cBos_Age24 -o hscr/diff_abund/taxa/cBos_P24_KW_L2.txt -m HSCR_diffabund_map.txt -i hscr/taxa/HSCR_OTU_table_L2.biom

group_significance.py -s nonparametric_t_test -c cBos_Age07 -o hscr/diff_abund/taxa/cBos_P07_NPT_L2.txt -m HSCR_diffabund_map.txt -i hscr/taxa/HSCR_OTU_table_L2.biom --permutations 1000 
group_significance.py -s nonparametric_t_test -c cBos_Age20 -o hscr/diff_abund/taxa/cBos_P20_NPT_L2.txt -m HSCR_diffabund_map.txt -i hscr/taxa/HSCR_OTU_table_L2.biom --permutations 1000 
group_significance.py -s nonparametric_t_test -c cBos_Age24 -o hscr/diff_abund/taxa/cBos_P24_NPT_L2.txt -m HSCR_diffabund_map.txt -i hscr/taxa/HSCR_OTU_table_L2.biom --permutations 1000 

# fecal
group_significance.py -s kruskal_wallis -c fBos_Age07 -o hscr/diff_abund/taxa/fBos_P07_KW_L2.txt -m HSCR_diffabund_map.txt -i hscr/taxa/HSCR_OTU_table_L2.biom
group_significance.py -s kruskal_wallis -c fBos_Age20 -o hscr/diff_abund/taxa/fBos_P20_KW_L2.txt -m HSCR_diffabund_map.txt -i hscr/taxa/HSCR_OTU_table_L2.biom
group_significance.py -s kruskal_wallis -c fBos_Age24 -o hscr/diff_abund/taxa/fBos_P24_KW_L2.txt -m HSCR_diffabund_map.txt -i hscr/taxa/HSCR_OTU_table_L2.biom

group_significance.py -s nonparametric_t_test -c fBos_Age07 -o hscr/diff_abund/taxa/fBos_P07_NPT_L2.txt -m HSCR_diffabund_map.txt -i hscr/taxa/HSCR_OTU_table_L2.biom --permutations 1000 
group_significance.py -s nonparametric_t_test -c fBos_Age20 -o hscr/diff_abund/taxa/fBos_P20_NPT_L2.txt -m HSCR_diffabund_map.txt -i hscr/taxa/HSCR_OTU_table_L2.biom --permutations 1000 
group_significance.py -s nonparametric_t_test -c fBos_Age24 -o hscr/diff_abund/taxa/fBos_P24_NPT_L2.txt -m HSCR_diffabund_map.txt -i hscr/taxa/HSCR_OTU_table_L2.biom --permutations 1000 

# genus (L6)
# colon
group_significance.py -s kruskal_wallis -c cBos_Age07 -o hscr/diff_abund/taxa/cBos_P07_KW_L6.txt -m HSCR_diffabund_map.txt -i hscr/taxa/HSCR_OTU_table_L6.biom
group_significance.py -s kruskal_wallis -c cBos_Age20 -o hscr/diff_abund/taxa/cBos_P20_KW_L6.txt -m HSCR_diffabund_map.txt -i hscr/taxa/HSCR_OTU_table_L6.biom
group_significance.py -s kruskal_wallis -c cBos_Age24 -o hscr/diff_abund/taxa/cBos_P24_KW_L6.txt -m HSCR_diffabund_map.txt -i hscr/taxa/HSCR_OTU_table_L6.biom

group_significance.py -s nonparametric_t_test -c cBos_Age07 -o hscr/diff_abund/taxa/cBos_P07_NPT_L6.txt -m HSCR_diffabund_map.txt -i hscr/taxa/HSCR_OTU_table_L6.biom --permutations 1000 
group_significance.py -s nonparametric_t_test -c cBos_Age20 -o hscr/diff_abund/taxa/cBos_P20_NPT_L6.txt -m HSCR_diffabund_map.txt -i hscr/taxa/HSCR_OTU_table_L6.biom --permutations 1000 
group_significance.py -s nonparametric_t_test -c cBos_Age24 -o hscr/diff_abund/taxa/cBos_P24_NPT_L6.txt -m HSCR_diffabund_map.txt -i hscr/taxa/HSCR_OTU_table_L6.biom --permutations 1000 

# fecal
group_significance.py -s kruskal_wallis -c fBos_Age07 -o hscr/diff_abund/taxa/fBos_P07_KW_L6.txt -m HSCR_diffabund_map.txt -i hscr/taxa/HSCR_OTU_table_L6.biom
group_significance.py -s kruskal_wallis -c fBos_Age20 -o hscr/diff_abund/taxa/fBos_P20_KW_L6.txt -m HSCR_diffabund_map.txt -i hscr/taxa/HSCR_OTU_table_L6.biom
group_significance.py -s kruskal_wallis -c fBos_Age24 -o hscr/diff_abund/taxa/fBos_P24_KW_L6.txt -m HSCR_diffabund_map.txt -i hscr/taxa/HSCR_OTU_table_L6.biom

group_significance.py -s nonparametric_t_test -c fBos_Age07 -o hscr/diff_abund/taxa/fBos_P07_NPT_L6.txt -m HSCR_diffabund_map.txt -i hscr/taxa/HSCR_OTU_table_L6.biom --permutations 1000 
group_significance.py -s nonparametric_t_test -c fBos_Age20 -o hscr/diff_abund/taxa/fBos_P20_NPT_L6.txt -m HSCR_diffabund_map.txt -i hscr/taxa/HSCR_OTU_table_L6.biom --permutations 1000 
group_significance.py -s nonparametric_t_test -c fBos_Age24 -o hscr/diff_abund/taxa/fBos_P24_NPT_L6.txt -m HSCR_diffabund_map.txt -i hscr/taxa/HSCR_OTU_table_L6.biom --permutations 1000 

# HSCR-Laramie
# phylum (L2)
# colon
group_significance.py -s kruskal_wallis -c cLar_Age07 -o hscr/diff_abund/taxa/cLar_P07_KW_L2.txt -m HSCR_diffabund_map.txt -i hscr/taxa/HSCR_OTU_table_L2.biom
group_significance.py -s kruskal_wallis -c cLar_Age20 -o hscr/diff_abund/taxa/cLar_P20_KW_L2.txt -m HSCR_diffabund_map.txt -i hscr/taxa/HSCR_OTU_table_L2.biom
group_significance.py -s kruskal_wallis -c cLar_Age24 -o hscr/diff_abund/taxa/cLar_P24_KW_L2.txt -m HSCR_diffabund_map.txt -i hscr/taxa/HSCR_OTU_table_L2.biom

group_significance.py -s nonparametric_t_test -c cLar_Age07 -o hscr/diff_abund/taxa/cLar_P07_NPT_L2.txt -m HSCR_diffabund_map.txt -i hscr/taxa/HSCR_OTU_table_L2.biom --permutations 1000 
group_significance.py -s nonparametric_t_test -c cLar_Age20 -o hscr/diff_abund/taxa/cLar_P20_NPT_L2.txt -m HSCR_diffabund_map.txt -i hscr/taxa/HSCR_OTU_table_L2.biom --permutations 1000 
group_significance.py -s nonparametric_t_test -c cLar_Age24 -o hscr/diff_abund/taxa/cLar_P24_NPT_L2.txt -m HSCR_diffabund_map.txt -i hscr/taxa/HSCR_OTU_table_L2.biom --permutations 1000 

# fecal
group_significance.py -s kruskal_wallis -c fLar_Age07 -o hscr/diff_abund/taxa/fLar_P07_KW_L2.txt -m HSCR_diffabund_map.txt -i hscr/taxa/HSCR_OTU_table_L2.biom
group_significance.py -s kruskal_wallis -c fLar_Age20 -o hscr/diff_abund/taxa/fLar_P20_KW_L2.txt -m HSCR_diffabund_map.txt -i hscr/taxa/HSCR_OTU_table_L2.biom
group_significance.py -s kruskal_wallis -c fLar_Age24 -o hscr/diff_abund/taxa/fLar_P24_KW_L2.txt -m HSCR_diffabund_map.txt -i hscr/taxa/HSCR_OTU_table_L2.biom

group_significance.py -s nonparametric_t_test -c fLar_Age07 -o hscr/diff_abund/taxa/fLar_P07_NPT_L2.txt -m HSCR_diffabund_map.txt -i hscr/taxa/HSCR_OTU_table_L2.biom --permutations 1000 
group_significance.py -s nonparametric_t_test -c fLar_Age20 -o hscr/diff_abund/taxa/fLar_P20_NPT_L2.txt -m HSCR_diffabund_map.txt -i hscr/taxa/HSCR_OTU_table_L2.biom --permutations 1000 
group_significance.py -s nonparametric_t_test -c fLar_Age24 -o hscr/diff_abund/taxa/fLar_P24_NPT_L2.txt -m HSCR_diffabund_map.txt -i hscr/taxa/HSCR_OTU_table_L2.biom --permutations 1000 

# genus (L6)
# colon
group_significance.py -s kruskal_wallis -c cLar_Age07 -o hscr/diff_abund/taxa/cLar_P07_KW_L6.txt -m HSCR_diffabund_map.txt -i hscr/taxa/HSCR_OTU_table_L6.biom
group_significance.py -s kruskal_wallis -c cLar_Age20 -o hscr/diff_abund/taxa/cLar_P20_KW_L6.txt -m HSCR_diffabund_map.txt -i hscr/taxa/HSCR_OTU_table_L6.biom
group_significance.py -s kruskal_wallis -c cLar_Age24 -o hscr/diff_abund/taxa/cLar_P24_KW_L6.txt -m HSCR_diffabund_map.txt -i hscr/taxa/HSCR_OTU_table_L6.biom

group_significance.py -s nonparametric_t_test -c cLar_Age07 -o hscr/diff_abund/taxa/cLar_P07_NPT_L6.txt -m HSCR_diffabund_map.txt -i hscr/taxa/HSCR_OTU_table_L6.biom --permutations 1000 
group_significance.py -s nonparametric_t_test -c cLar_Age20 -o hscr/diff_abund/taxa/cLar_P20_NPT_L6.txt -m HSCR_diffabund_map.txt -i hscr/taxa/HSCR_OTU_table_L6.biom --permutations 1000 
group_significance.py -s nonparametric_t_test -c cLar_Age24 -o hscr/diff_abund/taxa/cLar_P24_NPT_L6.txt -m HSCR_diffabund_map.txt -i hscr/taxa/HSCR_OTU_table_L6.biom --permutations 1000 

# fecal
group_significance.py -s kruskal_wallis -c fLar_Age07 -o hscr/diff_abund/taxa/fLar_P07_KW_L6.txt -m HSCR_diffabund_map.txt -i hscr/taxa/HSCR_OTU_table_L6.biom
group_significance.py -s kruskal_wallis -c fLar_Age20 -o hscr/diff_abund/taxa/fLar_P20_KW_L6.txt -m HSCR_diffabund_map.txt -i hscr/taxa/HSCR_OTU_table_L6.biom
group_significance.py -s kruskal_wallis -c fLar_Age24 -o hscr/diff_abund/taxa/fLar_P24_KW_L6.txt -m HSCR_diffabund_map.txt -i hscr/taxa/HSCR_OTU_table_L6.biom

group_significance.py -s nonparametric_t_test -c fLar_Age07 -o hscr/diff_abund/taxa/fLar_P07_NPT_L6.txt -m HSCR_diffabund_map.txt -i hscr/taxa/HSCR_OTU_table_L6.biom --permutations 1000 
group_significance.py -s nonparametric_t_test -c fLar_Age20 -o hscr/diff_abund/taxa/fLar_P20_NPT_L6.txt -m HSCR_diffabund_map.txt -i hscr/taxa/HSCR_OTU_table_L6.biom --permutations 1000 
group_significance.py -s nonparametric_t_test -c fLar_Age24 -o hscr/diff_abund/taxa/fLar_P24_NPT_L6.txt -m HSCR_diffabund_map.txt -i hscr/taxa/HSCR_OTU_table_L6.biom --permutations 1000
```

##### Achievement unlocked. Proceed to: Pre\_R\_Code.html¶
